# Ubiquitination of the Dishevelled DIX domain blocks its head-to-tail polymerization

**DOI:** 10.1038/ncomms7718

**Published:** 2015-04-24

**Authors:** Julia Madrzak, Marc Fiedler, Christopher M. Johnson, Richard Ewan, Axel Knebel, Mariann Bienz, Jason W. Chin

**Affiliations:** 1 MRC Laboratory of Molecular Biology, Cambridge Biomedical Campus, Francis Crick Avenue, Cambridge CB2 0QH, UK; 2 MRC Protein Phosphorylation and Ubiquitylation Unit, College of Life Sciences, University of Dundee, Dow Street, Dundee DD1 5EH, UK

## Abstract

Dishevelled relays Wnt signals from the plasma membrane to different cytoplasmic effectors. Its signalling activity depends on its DIX domain, which undergoes head-to-tail polymerization to assemble signalosomes. The DIX domain is ubiquitinated *in vivo* at multiple lysines, which can be antagonized by various deubiquitinases (DUBs) including the CYLD tumour suppressor that attenuates Wnt signalling. Here, we generate milligram quantities of pure human Dvl2 DIX domain mono-ubiquitinated at two lysines (K54 and K58) by genetically encoded orthogonal protection with activated ligation (GOPAL), to investigate their effect on DIX polymerization. We show that the ubiquitination of DIX at K54 blocks its polymerization in solution, whereas DIX58-Ub remains oligomerization-competent. DUB profiling identified 28 DUBs that cleave DIX-ubiquitin conjugates, half of which prefer, or are specific for, DIX54-Ub, including Cezanne and CYLD. These DUBs thus have the potential to promote Dvl polymerization and signalosome formation, rather than antagonize it as previously thought for CYLD.

## Abstract (web summary)

The relaying of Wnt signals to the cytoplasm requires the formation of signalosomes through the reversible polymerization of Dishevelled (Dvl). Here the authors establish the functional consequences of ubiquitination of the Dvl DIX domain and identify deubiquitinases predicted to promote Dvl polymerization.

## Graphical abstract

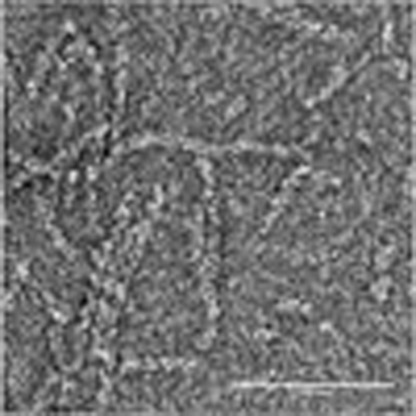

Wnt signals through β-catenin to control key steps in animal development and tissue homeostasis, and inappropriate activation of β-catenin leads to a range of human diseases, most notably cancer^1^. In the absence of Wnt, β-catenin is phosphorylated by glycogen synthase kinase 3 (GSK3) within the Axin destruction complex (‘Axin degradasome'^2^) that also contains the Adenomatous Polyposis Coli tumour suppressor, which earmarks β-catenin for ubiquitination and subsequent proteasomal degradation^3^. Binding of Wnt ligands to Frizzled receptors triggers recruitment of Dishevelled (Dvl) to the plasma membrane where Dvl assembles a signalosome to phosphorylate the cytoplasmic tail of the LRP5/6 co-receptor^4^. Signalosome formation by Dvl depends on head-to-tail polymerization by its DIX domain^5^,^6^, which also hetero-polymerizes with the DIX domain of Axin^7^, thereby recruiting Axin degradasomes to the plasma membrane^8^. Consequently, the phosphorylation of β-catenin by the degradasome-associated GSK3 is blocked, likely through direct inhibition by phosphorylated LRP6 tail, which binds to the catalytic site of GSK3 as a competitive inhibitor^9^. Hence, β-catenin accumulates and binds to T cell factor/lymphoid enhancer-binding factor (TCF/LEF) transcription factors in the nucleus, to operate a transcriptional switch^10^ that determines cell fates in both normal and diseased tissue contexts.

Dvl is a pivotal factor in Wnt signal transduction, relaying Wnt signals to canonical and non-canonical downstream effectors^3^. It contains three domains (separated by long flexible linkers) through which it binds to its signalling partners, typically with weak affinity^6^. These weak interactions depend on dynamic and concentration-dependent polymerization by the DIX domain^5^, which transiently generates high local concentrations of binding sites. This increases the avidity of Dvl for its low-affinity signalling partners, enabling their binding at low cellular concentration^6^. Polymerization of purified DIX domain can be monitored directly by biophysical methods^5^,^7^,^11^ and generates filaments that are detectable by electron microscopy^5^. In cells, DIX-dependent polymerization is essential for the activity of Dvl and Axin, enabling them to assemble signalosomes and degradasomes, respectively^2^,^7^. These are detectable as discrete puncta in cells that are highly dynamic, and rapidly exchanging with soluble protein^12^, in contrast to the irreversible fibrous aggregates that are formed by proteins such as α-synuclein that cause neurodegenerative disease^13^. Notably, the DIX domain is dedicated to the Wnt pathway and exclusively found in Dvl and Axin relatives, but is structurally related to the PB1 domain, which is found in other signalling molecules and which can also undergo head-to-tail polymerization^6^. All known DIX domain crystals contain helical filaments, whereby the same residues in the head and tail surfaces of DIX monomers are required for homo-polymerization of Dvl or Axin^5^,^11^ and for hetero-polymerization between Dvl and Axin^7^.

Interestingly, the DIX domain of mammalian Dvl1 has been found to be ubiquitinated *in vivo* at a highly conserved lysine residue (K50) and, depending on the conditions, also at other lysines (including K46) that serve as attachment sites for lysine 11-, 48- and 63-linked ubiquitin chains (K11-Ub, K48-Ub, K63-Ub)^14^,^15^,^16^. These ubiquitin chains can be trimmed by deubiquitinases (DUBs) of the large ubiquitin-specific protease (USP) family whose members typically cleave all ubiquitin linkages indiscriminately^17^, but also by the Cylindromatosis tumour suppressor CYLD^15^ whose narrow specificity for K63-Ub^18^ is somewhat unusual for this DUB family. Notably, the DIX domain is hyperubiquitinated in CYLD-depleted cells, and this was linked to enhanced signalling activity of Dvl1, which could be responsible for the growth of benign skin tumours such as cylindromas that arise in patients with germ-line mutations in CYLD^15^. However, it was unclear why the signalling activity of Dvl should be stimulated by DIX ubiquitination, especially since a Dvl1 mutant with a lysine-free DIX domain remained competent to assemble signalosomes^15^. More recently, USP14 was reported to trim K63-linked Ub chains attached to Dvl and promotes its signalling activity^16^, and the ubiquitination of the DIX domain within Dvl1 was reported to attenuate its self-association *in vivo*^14^. These authors therefore proposed^14^ that ubiquitination antagonized DIX-dependent polymerization, but this was not directly assessed in their study.

Despite a body of important *in vivo* work, it remains unclear (i) how ubiquitination at specific sites of the DIX domain affects its physical property of forming head-to-tail polymers and (ii) whether DUBs are able to cleave the isopeptide bonds between specific lysines in the DIX domain and the ubiquitin that is directly attached to them. To address these two questions, we generated recombinant DIX domain of human Dvl2 (hDvl2), with mono-Ub attached to either K54 or K58 (corresponding to K46 and K50 of mammalian Dvl1, the two DIX ubiquitination target sites in this Dvl protein uncovered by two independent studies^14^,^15^). As we intended to test these DIX-Ub conjugates in DUB assays, it was imperative to create native isopeptide bonds between ubiquitin and DIX, which is challenging to achieve by enzymatic methods. We thus adopted a strategy named GOPAL (genetically encoded orthogonal protection with activated ligation) that was developed to ubiquitinate recombinant proteins after co-translational incorporation of *N*ɛ-(*t*-butyloxycarbonyl)-L-lysine (referred to as Boc-lysine or denoted **1**) at specific sites^19^. This method was first applied to synthesize ‘atypical' ubiquitin dimers linked through K6 or K29, for DUB profiling and enabled the first K6 linked ubiquitin structure determination^19^. GOPAL was further refined by the application of Alloc-protecting groups in place of carboxybenzyl-protecting groups^20^ and used for the generation of ubiquitin chains of defined linkages and lengths^20^,^21^ as well as ubiquitination of the ubiquitin-like protein Rub1 (NEDD8 in mammalian cells)^21^.

Here, we successfully apply the refined GOPAL approach^19^,^20^ to synthesize milligram quantities of the hDvl2 DIX domain bearing site-specific and quantitative ubiquitination at either K54 (DIX54-Ub) or K58 (DIX58-Ub). We demonstrate that DIX54-Ub can only dimerize, whereas DIX58-Ub remains oligomerization-competent. Profiling a large panel of DUBs for their activity in removing ubiquitin from the DIX domain, we find that most USPs are capable of cleaving both DIX-Ub conjugates equally well, consistent with their broad activities against different ubiquitin linkages^17^. However, we also identify 14 DUBs with a clear preference or specificity towards cleaving DIX54-Ub, including highly specific DUBs such as CYLD and Cezanne, whose only previously known substrates are K63- and K11-linked ubiquitin, respectively^18^,^22^. Indeed, all but 6 of the 33 tested DUBs are active towards DIX54-Ub, implying that most DUBs are intrinsically capable of promoting Dvl polymerization.

## Results

### Site-specific ubiquitination of the hDvl2 DIX domain

**Figure 1:**
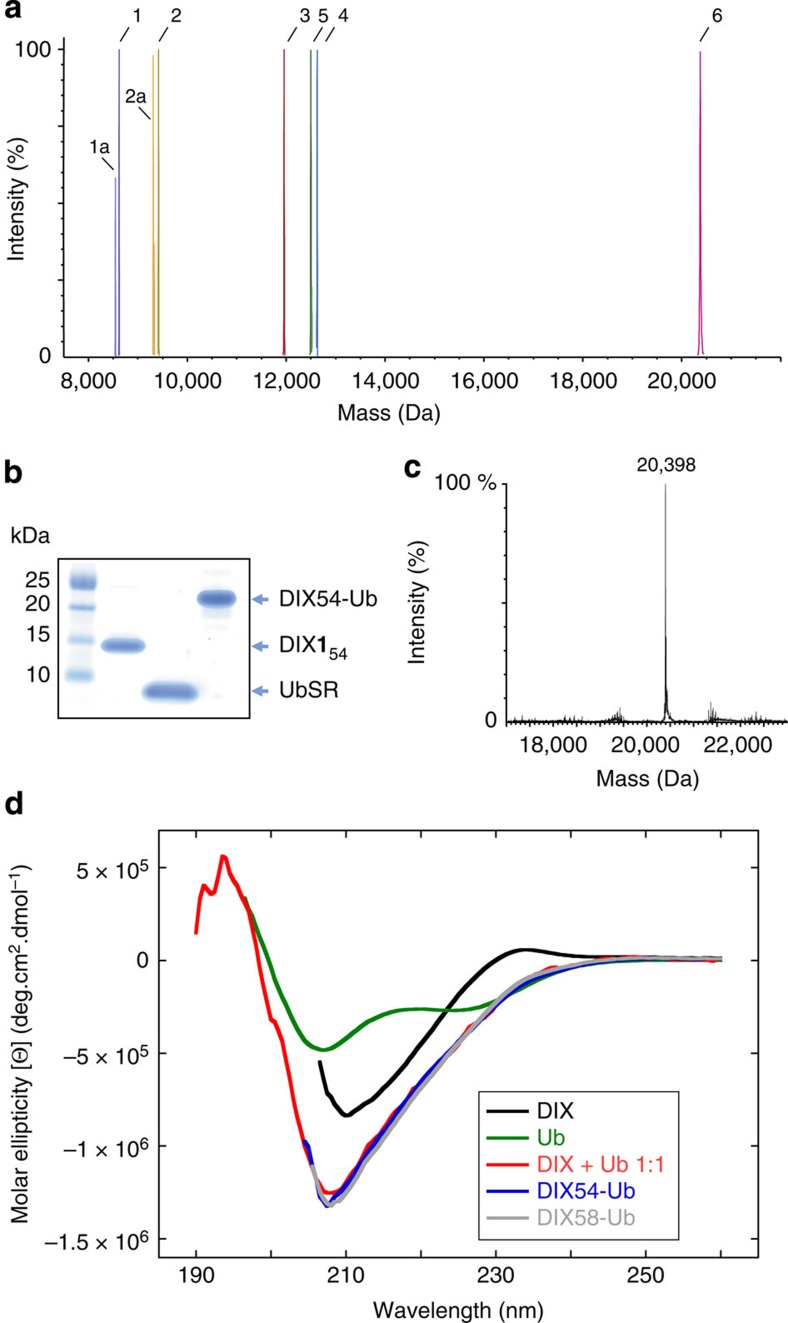
Synthesis and characterization of DIX-Ub conjugates. (**a**) Overlay of LC-MS traces during synthesis of DIX54-Ub; violet (1), UbSR, purified ubiquitin-MES thioester (found 8,688 Da, calculated 8,689 Da); lilac (1a), hydrolysed UbCOOH (observed 8,564 Da, found 8564 Da); ochre (2), UbSR(9Alloc), UbSR after chemical Alloc protection (observed 9,445 Da, calculated 9,445 Da); yellow (2a), UbCOOH(9Alloc), UbCOOH after chemical Alloc protection (observed 9,231 Da, calculated 9,230 Da); red (3), DIX**1**_54_, DIX with **1** genetically incorporated at K54 (observed 11,948 Da, calculated 11,948 Da); blue (4), DIX**1**_54_(8Alloc), DIX**1**_54_ after chemical protection with Alloc (observed 1,260 Da, calculated 1,260 Da); green (5), DIXK_54_(8Alloc), DIX**1**_54_(8Alloc) after Boc deprotection (observed 12,520 Da, calculated 12,520 Da); pink (6), DIX54-Ub, DIX54-Ub after Alloc deprotection (observed 20,394 Da, calculated 20,395 Da); **1** is *Nɛ*-(*t*-butyloxycarbonyl)-L-lysine. These are deconvoluted spectra, all spectra are shown before deconvolution in Supplementary Figs 6–11. (**b**) SDS–polyacrylamide gel electrophoresis of starting materials and products for DIX54-Ub synthesis. (**c**) ESI-MS analysis of DIX54-Ub; observed mass=20,398 Da, expected mass=20,395 Da. (**d**) Overlaid CD spectra of DIX, Ub or equimolar mixtures of DIX+Ub, DIX54-Ub and DIX58-Ub. For the synthesis and characterization analysis of DIX58-Ub and other DIX-Ub conjugates, see Supplementary Figs 2–11.

We generated site-specifically ubiquitinated DIX domain from human hDvl2 using GOPAL^19^,^20^ (Supplementary Fig. 1), further modified as follows. We produced the DIX domain bearing *Nɛ*-(*t*-butyloxycarbonyl)-L-lysine at position 54 and an *N*-terminal His_6_-tag followed by a TEV protease cleavage site (His_6_-DIX**1**_54_, Supplementary Table 1 for protein sequences) from *E. coli* expressing the *Methanosarcina barkeri* pyrrolysine tRNA synthetase (*Mb*PylRS)/*Mb*tRNA_CUA_ pair, and His_6_-DIX(54TAG) encoding a hexahistidine tag followed by a TEV cleavage site, and the DIX domain of hDvl2 bearing an amber codon at position 54. His_6_-DIX**1**_54_ was purified by Ni-NTA chromatography with a yield of 2–3 mg per l of culture. The His_6_-tag was removed with TEV protease, and the resulting DIX**1**_54_ protein was further purified by anion exchange and size-exclusion chromatography (Supplementary Fig. 2). Efficient incorporation of Boc-lysine into DIX was confirmed by electrospray ionization mass spectrometry (Fig. 1a).

We protected the nucleophilic amines in DIX-**1**_54_ with allyloxycarbonyl (Alloc) groups, creating DIX**1**_54_(8Alloc). Selective removal of the Boc group with 60% trifluoroacetic acid (TFA) led to DIXK_54_(8Alloc), bearing a single unprotected lysine side chain at position 54 of DIX. Ubiquitin bearing a C-terminal thioester (UbSR), generated by MESNa-induced thiolysis of a recombinant ubiquitin-intein fusion^23^,^24^, was protected with Alloc-OSu, creating UbSR(9Alloc). To create the isopeptide bond between DIX K54 and the C-terminus of ubiquitin, we reacted the single free amine in DIXK_54_(8Alloc) with UbSR(9Alloc) in a Ag(I) catalysed condensation^23^. The Alloc-protecting groups were removed with chloro-pentamethylcyclopentadienyl-cyclooctadiene-ruthenium(II) ([Cp*Ru(cod)Cl], 100% mol)^25^, and DIX54-Ub was purified by denaturing size-exclusion chromatography. After slow renaturation by dialysis against PBS buffer, the protein was purified further by non-denaturing size-exclusion chromatography. Using this approach, we generated 1–2 mg of DIX54-Ub from 10 mg of DIX**1**_54_ (Fig. 1b). We repeated the procedure using His_6_-DIX(58TAG) in place of His_6_-DIX(54TAG), to create DIX58-Ub in comparable yield (Supplementary Fig. 3). Both DIX54-Ub and DIX58-Ub had the expected mass, and electrospray ionization tandem mass spectrometry (ESI MS)-MS confirmed the isopeptide linkage at the genetically programmed site with no isopeptide linkages detected at non-programmed sites (Fig. 1c and Supplementary Figs 4–11). The circular dichroism (CD) spectra of DIX54-Ub and DIX58-Ub are comparable to the CD spectra of an equimolar mixture of DIX and ubiquitin, indicating normal folding of the two DIX-Ub conjugates (Fig. 1d).

### K54-Ub blocks DIX polymerization in solution

**Figure 2:**
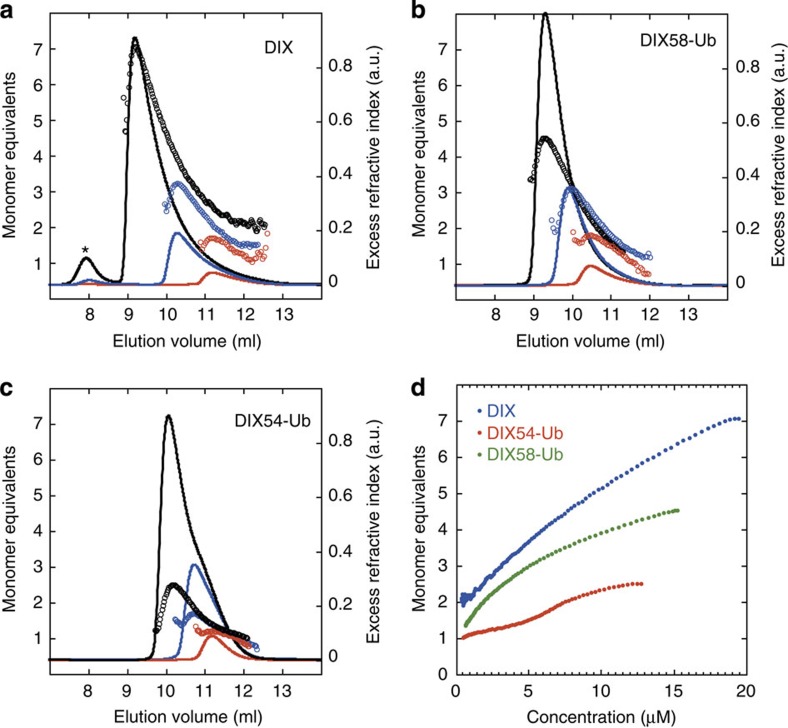
K54-Ub blocks DIX polymerization in solution. SEC-MALS analysis of (**a**) DIX, (**b**) DIX58-Ub or (**c**) DIX54-Ub at different starting concentrations; open circles indicate molecularity (apparent molecular mass calculated from MALS/molecular weight of proteins; *black*, 235 μM DIX, or ∼160 μM DIX54-Ub or DIX58-Ub; *blue*, 50 μM; *red*, 10 μM; note that these initial sample concentration were reduced by ∼10x during the SEC step and at subsequent MALS and RI detection); solid lines, overall elution profile as detected by the refractive index detector. (**d**) Summary of SEC-MALS for the highest concentration data points (DIX, 235 μM; DIX54-Ub, <160 μM; DIX58-Ub, 160 μM). The degree of polymerization was quantified by SEC-MALS, based on measurements of scattering intensity and refractive index signal as concentration source (d*n*/d*c* of 0.186 ml g^−1^), as shown in Fig. 2a–c. Masses were transformed into number of monomer equivalents in the polymer, based on the molecular weight of each DIX protein (plus Ub conjugate where present), and the number of monomer equivalents was recast against molar concentration (rather than elution volume) based on the concentration determined from refractive index signals and known molecular masses.

We used size exclusion chromatography coupled to multi-angle light scattering (SEC-MALS) as a quantitative assay to determine the effects of K54- and K58-ubiquitination on DIX domain polymerization in solution. Unmodified DIX domain undergoes concentration-dependent and reversible polymerization^5^, forming some large protein assemblies that elute as a first peak in the SEC-MALS chromatogram (Fig. 2a, asterisk). These large DIX assemblies are polydisperse, and we estimate their molecular mass to be >1 MDa (corresponding to >84 monomers). The radius of gyration of this material was ≈20 nm and considerably larger than the measured hydrodynamic radius, thus consistent with an extended and non-globular conformation. This material may thus contain short DIX filaments (as previously observed by electron microscopy^5^). This first peak is consistently absent in the DIX58-Ub and DIX54-Ub chromatograms (Fig. 2b,c), indicating that the filament assembly of these DIX-Ub conjugates is severely attenuated in solution. However, the bulk of the unmodified DIX domain (that is, 94–95% of the total protein, depending on the protein concentration; Fig. 2a) elutes as a major second peak corresponding to DIX oligomers whose molecular mass can be determined accurately, as described below.

To quantify the concentration-dependent polymerization of unmodified and ubiquitinated DIX, we conducted SEC-MALS at different initial concentrations of DIX and DIX-Ub. At a loading concentration of 10 μM unmodified DIX, the largest species formed has an average molecular weight corresponding to a DIX dimer (Fig. 2a, red), whereas DIX forms an apparent trimer at 50 μM (Fig. 2a, blue) and a hexamer or heptamer at the highest concentration at which this domain remained in solution (235 μM; Fig. 2a, black), consistent with previous results from equilibrium ultracentrifugation^5^. DIX58-Ub behaves similarly, forming an apparent trimer at 50 μM, and an apparent tetramer or pentamer at the highest concentration (Fig. 2b, black). In contrast, DIX54-Ub can barely form a dimer, even at the highest concentration (Fig. 2c, black). We conclude that ubiquitination at DIX K54 strongly interferes with the head-to-tail interaction between DIX monomers, thereby suppressing polymerization in solution, whereas K58 ubiquitination has a much weaker effect as DIX58-Ub remains capable of forming oligomers.

Quantification of these SEC-MALS data (based on scattering intensity, refractive index signals and molecular mass of DIX or DIX-Ub conjugates recast against molar concentration) confirms that unmodified DIX can form up to heptamers at high concentrations, similarly DIX58-Ub reaches tetramers or pentamers at comparable concentrations, whereas DIX54-Ub apparently plateaus at dimers (Fig. 2d, red). This confirms that the ubiquitination of DIX at K54 essentially blocks its polymerization in solution, whereas DIX58-Ub remains oligomerization-competent, although its polymerization into filaments seems to be blocked.

### hDvl2 K54 projects into the DIX-DIX interface

**Figure 3:**
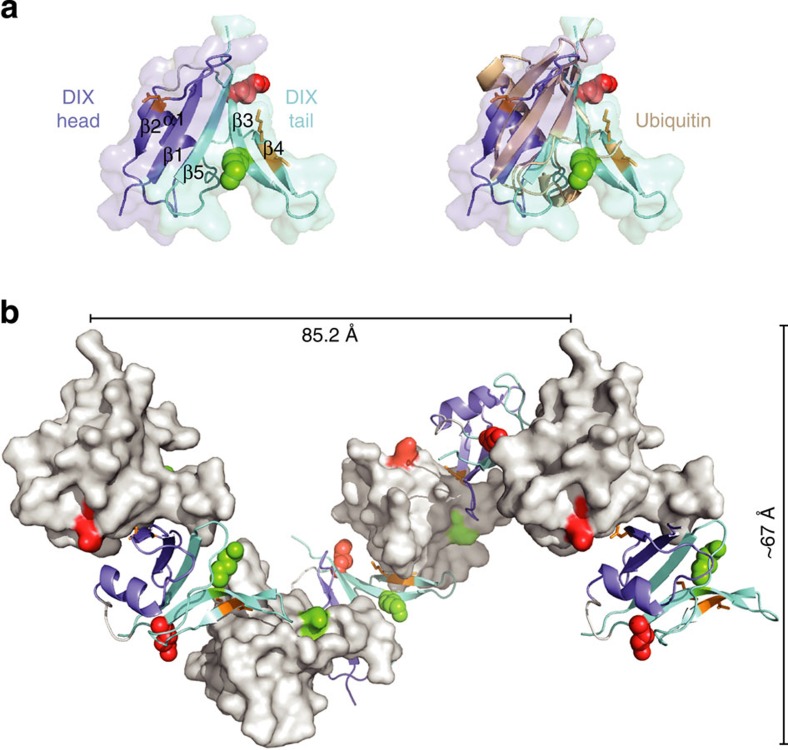
Crystal structure of the hDvl2 DIX domain. (**a**) Ribbon representation of the hDvl2 DIX monomer structure (4WIP; head, *purple*; tail, *cyan*), and overlay with ubiquitin (1UBQ; *wheat*), to reveal the similarity between their overall folds; K54 (red) and K58 (green) are shown in sphere representation. (**b**) Structure and dimensions of the DIX filament (with alternate monomers in ribbon or surface representation), with colours as in **a**; residues mediating key contacts in the DIX–DIX interfaces (V67 K68, mutated in M2; Y27, mutated in M4) are indicated in stick representation (orange).

To understand the structural basis for the polymerization block imposed by DIX54-Ub, we purified the hDvl2 DIX domain (bearing Y27D; see Methods), and solved its crystal structure at 2.7 Å resolution (Table 1). Like other DIX domains^5^,^11^, the hDvl2 DIX monomer exhibits a ubiquitin-like fold, with five β-strands and an α-helix (Fig. 3a). Its structure is very similar to that of the Dvl1 DIX monomer^11^ (with a root-mean-square deviation (r.m.s.d). for the core Cα backbone of 1.14 Å between hDvl2 MolA and Dvl1 MolF), whereby the main differences are in two loop regions (between β1 and β2, and α1 and β3; Supplementary Fig. 12). There are marked differences in the dimensions of the DIX filament structures in the crystals: the filament of hDvl2 DIX is ∼67 Å wide, and its repeat length is 85 Å, with 6 monomers per turn (Fig. 3b), almost identical to the Axin DIX filament^5^, whereas the filament in the Dvl1 DIX domain crystal (from the equivalent mutant, Y17D) is nearly twice as long as the Axin DIX filament, with a repeat length of ∼140 Å and 8 monomers per turn^11^. As the helical pitch determines the packing of adjacent filaments in the crystals, it is unsurprising that the two types of DIX filaments contact each other at different sites (termed site III)^11^, but it seems likely that these contacts, like the filamental dimensions, simply reflect the different crystallization conditions. We note also that site III mutations (that do not affect the nearby tail surface) neither suppress DIX polymerization nor Dvl signalling activity^11^.

hDvl2 K54 and K58 are both located within the DIX tail portion (Fig. 3b): K54 lies at the junction between β3 and the preceding loop, flanking the DIX–DIX interface and pointing towards this interface (roughly parallel to the M2 residue V67), whereas K58 is within β3, but points away from this interface into the solvent. This predicts that a Ub moiety attached to K54 would create a steric clash with the head surface of an interacting DIX monomer, whereas one attached to K58 would be oriented perpendicularly to the filamental axis and thus not impinge on the DIX–DIX interaction (although we note that ubiquitin contains an extended flexible C-terminus, and may thus be able to adopt multiple conformations relative to the DIX domain). These structural predictions are borne out by our SEC-MALS data (Fig. 2) showing that polymerization in solution is suppressed by K54-Ub, whereas K58-Ub remains competent to form oligomers. Given that K58 projects into the inside of the helical filament (Fig. 3b), stoichiometric ubiquitination of this residue might affect the filamental shape, which could explain why this modification limits oligomerization to a single helical pitch in solution (Fig. 2d).

### DIX filament assembly is severely attenuated by K54-Ub

**Figure 4:**
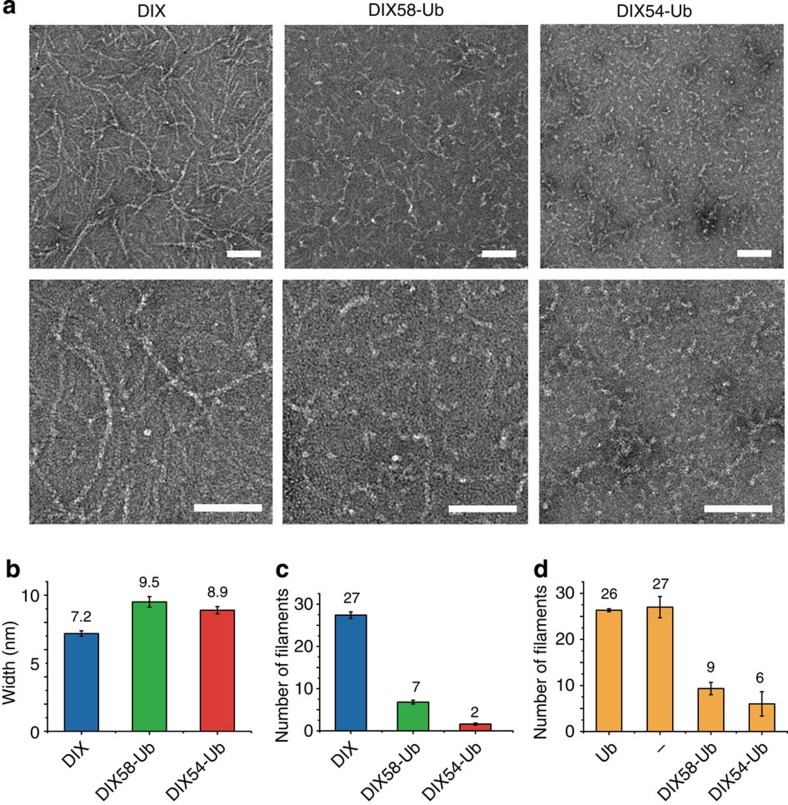
K54-Ub severely attenuates DIX filament assembly. (**a**) Transmission electron microscope images of 100 μM unmodified DIX or DIX-Ub conjugates, as indicated above panels, at two different magnifications (*top*, × 15,000; *bottom*, × 30,000); scale bars, 100 nm (see also Supplementary Fig. 13). (**b**) Mean widths of DIX and DIX-Ub filaments; five representative filaments were selected from different micrographs, and their width was measured at five random locations (*n*=25). (**c**) Mean number of filaments (>100 nm) per micrograph (*n*=10), as shown in **a** *top*. (**d**) Mean number of DIX filaments determined as in **c**, following mixing (at 2:1) of DIX with Ub, buffer (-), DIX58-Ub or DIX54-Ub, as indicated; error bars, standard errors.

We used electron microscopy of negatively stained DIX domain, to monitor the effects of its ubiquitination on the formation of DIX filaments. The unmodified DIX domain readily forms long, well-defined filaments^5^ (Fig. 4a), with a consistent mean width of ∼7 nm (Fig. 4b). This corresponds to the width determined for a single DIX filament in the crystal (Fig. 3b), so the filaments that are observed by electron microscopy most likely correspond to DIX proto-filaments. Their length varies considerably, ranging from 150 to 800 nm (that is, reaching up to 100 helical turns; Supplementary Fig. 13). These filaments have a tendency to coalesce into fibres composed of multiple proto-filaments, <100 nm wide (Supplementary Fig. 13), but the incidence of this is difficult to quantify as it varies greatly between preparations and different positions on the electron microscope grids (as fibre formation is likely to be triggered stochastically by high DIX concentrations during the absorption of the sample onto the grid). Importantly, neither proto-filaments nor fibres are observed with polymerization-deficient DIX domains^5^.

DIX58-Ub can also form filaments, but these are far less abundant than those formed by unmodified DIX (Fig. 4c). Furthermore, the DIX58-Ub filaments often look distorted (Fig. 4a) and have a slightly wider diameter (∼9.5 nm; Fig. 4b). This is consistent with the structural predictions that ubiquitination at K58, although compatible with filament formation, would affect the filamental shape. Somewhat surprisingly, filaments can even be detected in DIX54-Ub preparations, but these are very rare, and they also look abnormal (Fig. 4a–c), like the DIX58-Ub filaments. However, neither of the DIX-Ub samples show fibres, presumably because of the low concentration of filaments in these samples and their abnormal shapes (which could prevent lateral coalescence). Evidently, the conditions of these electron microscopy assays are more permissive for filament assembly by DIX-Ub conjugates than those of the SEC-MALS assays (in which DIX-Ub filaments were undetectable as judged by the lack of the first peaks in the chromatographs shown in Fig. 2b,c). The reason for this slight discrepancy between the two assays is likely to reflect mainly the differences in protein concentrations, but also the sensitivity of detection: the DIX domain is applied to electron microscope grids at 100 μM and becomes even more concentrated when drying, which could trigger filament assembly, while its initial concentration in the SEC-MALS assays is ∼5 × lower (and the samples become gradually more diluted during the gel chromatography). The SEC-MALS assay thus provides a realistic and quantitative reflection of DIX oligomerization in solutions of low protein concentrations, but is less sensitive in detecting DIX filaments.

Next, we asked whether ubiquitination of DIX acts dominant-negatively on filament formation by unmodified DIX. We thus spiked DIX with free ubiquitin, or with DIX54-Ub or DIX58-Ub, at defined ratios of 10:1 (which had no detectable effect) or 2:1, which reduced the frequency of DIX filaments by >4 × (DIX54-Ub) or by ∼3 × (DIX58-Ub; Fig. 4d). As these dominant-negative activities of DIX-Ub can be observed under conditions where unmodified DIX is in excess, this implies that sub-stoichiometric ubiquitination of the DIX domain can attenuate its ability to polymerize.

### Identifying DUBs that cleave the isopeptide bond of DIX-Ub

Several DUBs have been shown to affect Wnt signalling, apparently through targeting components of the Dvl signalosomes and their interacting proteins—including CYLD^15^, USP14 (ref. 16), USP8 (ref. 26) and Trabid^27^. These DUBs have been tested for their activity towards different types of ubiquitin linkages, but it is unknown whether they can remove ubiquitin from target proteins. DUBs that remove ubiquitin from DIX54-Ub, rather than simply cleaving isopeptide bonds between Ub monomers in the Ub chains attached to the DIX domain, are predicted to alleviate the polymerization block imposed by this ubiquitination and, thus, to promote signalosome assembly by Dvl.

We used *in vitro* DUB assays^19^,^28^,^29^, to identify DUBs that cleave the isopeptide bond in DIX-Ub conjugates, testing 33 different DUBs for their ability to generate free ubiquitin and DIX alongside appropriate diUb controls. These 33 enzymes represent >1/3 of all known human DUBs, and they include members of all four major DUB families—20 USPs (including CYLD, USP14 an USP8), 8 Ovarian Tumour (OTU) deubiquitinases (including Trabid and Cezanne), 3 ubiquitin C-terminal hydrolases (UCHs) and 2 JAMM metalloproteases. To avoid complicating effects due to the DIX polymerization in these assays, we also synthesized and purified polymerization-defective mutants of DIX54-Ub and DIX58-Ub bearing the M2 mutation (V67A K68A)^5^.

**Figure 5:**
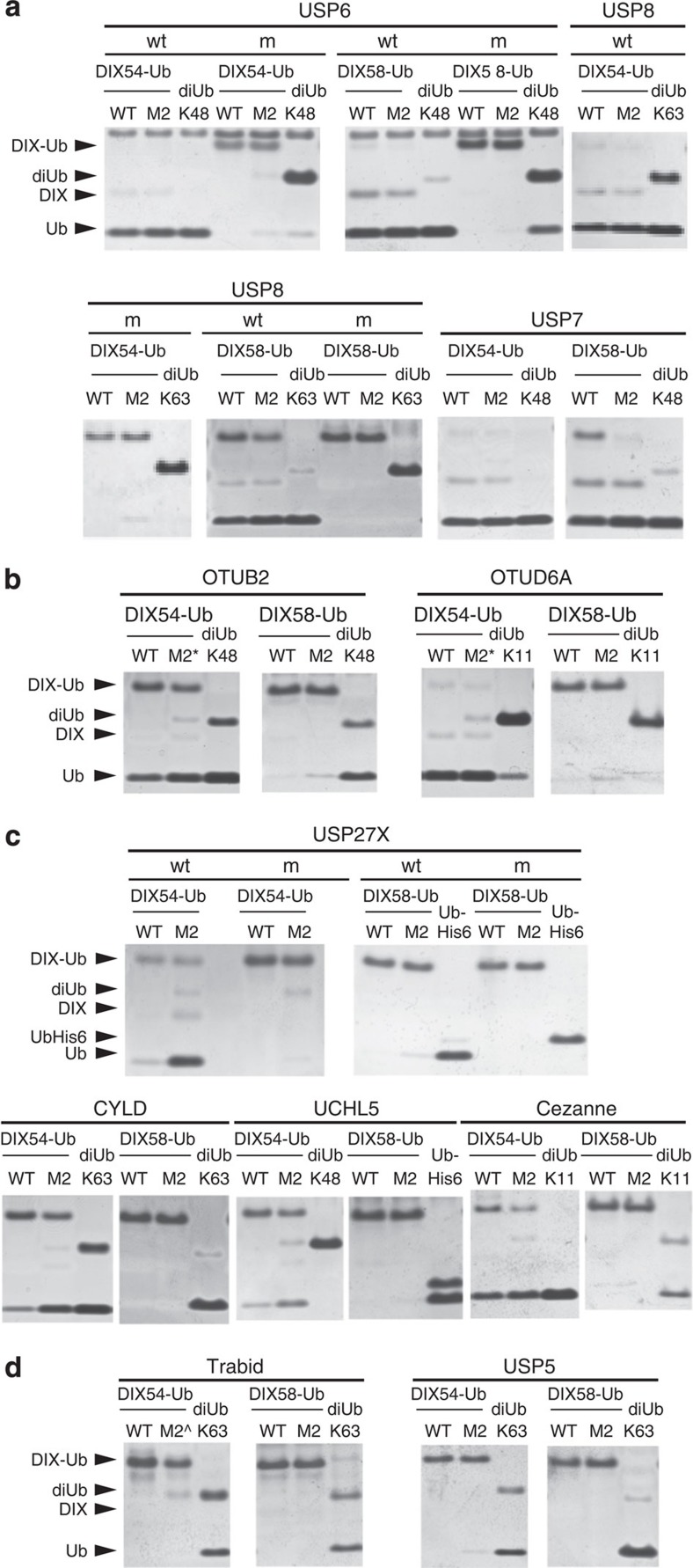
DUB profiling reveals preference for DIX54-Ub over DIX58-Ub. Assays with purified recombinant DUBs from the USP, OTU, UCH and JAMM metalloprotease families, after incubation for 60 min with DIX-Ub conjugates, and various diUb (or Ub-His_6_) as controls, as indicated above panels; wt, wild-type DUB; m, catalytically dead DUB. Representative examples are shown of (**a**) group 1, (**b**) group 2, (**c**) group 3 and (**d**) group 4 (see Table 2). See also Supplementary Fig. 14, for full-length gels and assays of additional DUBs.

Each of the USPs proved to be active against at least one DIX-Ub, except for USP5 (Fig. 5 and Table 2), which has a rather unusual specificity for cleaving long unanchored polyUb chains^30^, and USP14 (Supplementary Fig. 14 and Table 2), which also proved to be inactive against diUb, as expected since the activity of this DUB on diUb depends on its association with the proteasome^31^,^32^. Of the 18 active USPs, 13 were equally active against DIX54-Ub and DIX58-Ub (including USP8; Fig. 5a). The remaining USPs displayed either a strong preference (USP10 and USP28) for DIX54-Ub over DIX58-Ub (Fig. 5b), or specificity (CYLD, USP25, USP27X) for cleaving only DIX54-Ub (Fig. 5c). The same was observed if the polymerization-deficient mutants were used as DUB substrates (Supplementary Fig. 14). Clearly, most USPs are active towards both DIX-Ub conjugates, although some show preference or specificity towards DIX54-Ub. This is consistent with their indiscriminate activity against different ubiquitin linkages^17^.

In contrast, the OTUs proved to be far more selective in our assays, as expected from their high degree of substrate selectivity towards different ubiquitin linkages^29^. Remarkably, none of the eight tested OTUs efficiently cleaved DIX58-Ub: four of them preferred DIX54-Ub, Cezanne was specific for DIX54-Ub and the remaining three (OTUB1, A20, Trabid) cleaved neither, although they were all active in cleaving diUb controls (Fig. 5b–d; Table 2). Similarly, the UCHs and JAMM metalloproteases displayed a high degree of specificity, with UCHL5, BAP1 and AMSH being only active towards DIX54-Ub. Only one member of these two groups (UCHL3) cleaved both types of DIX-Ub indiscriminately (Fig. 5c; Table 2 and Supplementary Fig. 14). In summary, the members of these three DUB families either prefer DIX54-Ub over DIX58-Ub as a substrate, are specific for DIX54-Ub, or cannot cleave either DIX-Ub conjugate, and thus show a high degree of substrate selectivity.

## Discussion

We applied a modified GOPAL strategy to generate milligrams of pure hDvl2 DIX domain site-specifically ubiquitinated at either K54 or K58, which allowed us for the first time to determine the direct effects of these ubiquitinations on the head-to-tail polymerization of the DIX domain by biophysical and microscopic methods. We thus found that polymerization of the DIX domain was essentially suppressed by ubiquitination at K54 and also reduced by ubiquitination at K58, broadly consistent with the proposal from a recent study^14^, based on indirect evidence, that ubiquitination of the DIX domain attenuates its polymerization. Our findings are also reminiscent of the results with α-synuclein, a presynaptic protein implicated in Parkinson's disease, whose self-assembly into fibrils was shown to be attenuated or blocked by ubiquitylation at specific sites^33^, based on semi-synthetic approaches of generating well-defined α-synuclein-Ub conjugates^33^,^34^,^35^. Furthermore, by profiling a large panel of DUBs for their activities towards our DIX-Ub conjugates, we identified representatives of all four major DUB families that cleave DIX54-Ub, which thus have the potential to promote the DIX-dependent polymerization by which hDvl2 assembles signalosomes in cells. To our knowledge, this is the first example of a large-scale profiling of DUBs for their activity against distinct native isopeptide bonds between ubiquitin and a target protein domain.

Clearly, the two ubiquitinations of the DIX domain have different effects on its polymerization and filament assembly: K54-Ub severely interferes with DIX polymerization, allowing DIX merely to dimerize if assayed in solution (that is, by SEC-MALS), and K54-Ub filaments are barely detectable by electron microscopy. By contrast, ubiquitination at DIX K58 is compatible with DIX polymerization as DIX58-Ub efficiently oligomerizes up to one helical pitch in solution. This may explain why K58-Ub forms filaments more readily than K54-Ub in the permissive electron microscopy assay, although in both cases, these filaments are distorted. Our results can be rationalized by the crystal structure of the DIX domain: K54 points into the DIX–DIX interface, and its ubiquitination is predicted to clash with the interacting DIX monomer, whereas K58 points away from this interface, roughly perpendicularly to the filamental axis. Even though K58 projects into the inside the DIX filament, the linked ubiquitin is attached through its flexible C-terminus, allowing it to be accommodated by the DIX filament whose extended conformation may be able to absorb multiple ubiquitin moieties per helical pitch without major structural distortion. An important corollary of our findings is that ubiquitination at K54 of hDvl2 would inhibit signalosome assembly *in vivo* and Wnt signal transduction. Indeed, according to structural predictions, this is the only ubiquitination of Dvl2 DIX with the potential to block polymerization—apart from K68-Ub, which is bound to block polymerization as K68 is located in the tail surface and directly participates in the DIX–DIX interaction domains^5^,^11^. By contrast, the oligomerization of K58-ubiquitinated DIX (which remains efficient up to one helical pitch) might suffice for signalling activity, as even short oligomers should confer a significant increase in avidity on Dvl for low-affinity ligands^6^. Interestingly, the latter modification is the only ubiquitination that has been detected so far in untreated cells (that is, in the absence of Wnt stimulation or other manipulations)^14^.

CYLD is a negative regulator of Wnt signalling, and its functionally relevant target in the Wnt pathway appears to be Dvl (ref. 15). These authors noted a close correlation between increased DIX ubiquitination, and increased polymerization and signalling activity of Dvl, in CYLD-deficient cells. However, our data are inconsistent with a direct causal relationship between these effects: we showed that K54-Ub blocks DIX-dependent polymerization, and that this modification is cleaved by CYLD. Therefore, the activity of this DUB should stimulate rather than attenuate the signalling activity of Dvl, which means that the observed downregulation of Wnt signalling by CYLD^15^ cannot be explained by its effect on Dvl polymerization. However, we note that the association of CYLD with Dvl1 destabilized Dvl1 (Fig. 4c in ref. 15), which could provide an alternative explanation for why Dvl signalling is increased in CYLD-deficient cells. Other explanations are also conceivable (for example, relating to CYLD's function in stabilizing astral microtubules, which involves an interaction with Dvl (ref. 36) that could affect its signalling activity).

Our DUB profiling revealed that virtually all tested USPs are candidates for promoting DIX-dependent polymerization and signalling by hDvl2: the majority hydrolysed both types of DIX-Ub conjugates equally well, consistent with their broad activity towards different Ub linkages^17^, although some USPs preferred cleaving DIX54-Ub over DIX58-Ub, and three (including CYLD) were specific for DIX54-Ub. USP8 belongs to the first group of DUBs that are active against both DIX-Ub conjugates, and this activity towards Dvl might therefore contribute to its stimulatory effect on Wnt signalling (proposed to reflect increased recycling of Fz receptor^26^). By contrast, the stimulatory effect of USP14 on Dvl signalling activity (reported to be proteasome-independent^16^) is less likely to involve the DIX domain as this DUB proved inactive against both DIX-Ub conjugates. However, there is an important caveat regarding the DUBs that lack activity in our assays (which are based on the isolated DIX domain): if these DUBs bind to Dvl through a sequence outside the DIX domain (as in the case of USP14 (ref. 16)), they may nevertheless be capable of deubiquitinating this domain in the context of full-length Dvl.

Interestingly, five of the eight tested OTU DUBs also proved to be active against our DIX-Ub conjugates although, in contrast to most USPs, they all displayed substrate preference or selectivity: half of the tested OTU enzymes strongly preferred DIX54-Ub over DIX58-Ub, and Cezanne specifically cleaved DIX54-Ub. These results were somewhat unexpected, in the light of the high specificity of this DUB class with regard to their substrates: indeed, the activity of Cezanne was previously reported to be limited to K11-Ub^22^, and OTUD3 and OTUB2 also cleave only a limited set of ubiquitin linkages, whereas OTUD2 and OTUD6A are somewhat broader^29^. Our results provide the first evidence that these DUBs can also remove ubiquitin from target proteins, and thus widen the spectrum of activities for this highly specific DUB family.

The remaining three OTU DUBs (A20, OTUB1 and Trabid) were inactive against both DIX-Ub conjugates and are candidates for negative regulators of Dvl polymerization. However, since they cleave K48-linked (A20, OTUB1)^28^,^29^ or K63-linked ubiquitin chains (Trabid)^19^,^27^,^29^, both of which are attached to Dvl during Wnt signalling^15^, they could increase the stability of Dvl by removing these chains, so might have a net positive effect on Wnt signalling, especially in the presence of USP8 or other USPs.

In summary, our DUB assays uncovered four groups of enzymes with distinct substrate specificities (Table 2). Remarkably, we found no enzyme with specificity (or preference) for DIX58-Ub. Thus, DIX58-Ub is only cleaved by DUBs without substrate specificity, whereas DIX54-Ub is an excellent substrate for a wide range of distinct DUBs.

Substrate recognition by DUBs remains poorly understood. However, the known co-crystal structures between DUBs and their native substrates have uncovered broadly two features that determine the specificity of DUBs towards their substrates^37^,^38^,^39^. In the complex between JAMM metalloprotease AMSH-LP and K63-diUb, the two residues immediately flanking the K63 attachment site of the proximal ubiquitin (that is, Q62 and E64) undergo crucial hydrogen bonds with the catalytic pocket of AMSH-LP, which may limit its activity towards K63-linked ubiquitin^38^. In DIX58-Ub, the corresponding residues are F57 and S59, and it is conceivable that one or both of these residues cannot be accommodated by the catalytic pockets of the DUBs (from groups 2 to 4) that cannot cleave this substrate.

The co-crystal structures of two OTU DUBs with their substrates have revealed a second feature that impacts on their specificity^37^,^39^: in each case, the DUB undergoes specific interactions with the proximal and distal ubiquitin, but this substrate recognition requires a certain overall conformation of the diUb, which is determined by the linkage between the two ubiquitins. Recall that the DIX domain has a ubiquitin-like fold^5^ (Fig. 3a), and it is possible that DIX54-Ub but not DIX58-Ub adopts a conformation that resembles certain native diUbs. Recall also that Cezanne only cleaves DIX54-Ub (Fig. 5c) and, of all ubiquitin linkages, only K11-diUb^22^,^29^. Indeed, the observed DIX54-Ub preference or specificity of the tested DUBs (Table 2) correlates with their ability to cleave K11-diUb (OTU DUBs in group 2)^29^ or K63-diUb (CYLD)^18^, but negatively correlates with specificity for K48-diUb (OTUB1, A20)^29^. Intriguingly, K63-diUb and K11-diUb adopt extended conformations^28^,^40^, whereas K48-diUb is relatively compact^41^. If DIX54-Ub were to adopt an extended conformation loosely resembling these extended diUbs (Supplementary Fig. 12), this might explain why DIX54-Ub is the preferred substrate for DUBs cleaving extended diUbs.

In conclusion, our DUB assays identified candidates for regulators of the DIX-dependent polymerization of Dvl that underlies its signalling activity, and enabled us to obtain insights into the rules and mechanisms governing Ub-protein cleavage. The results from these assays are conclusive because they are based on native isopeptide bonds in the DIX-Ub substrates. Previous studies of conjugates between ubiquitin and non-Ub targets include Rub1 (ref. 21), histones^42^,^43^,^44^, proliferating cell nuclear antigen (PCNA)^45^ or α-synuclein^34^, but some of these were generated by chemical ligation methods that resulted in non-native bonds between ubiquitin and target, which limits the utility of these conjugates for DUB profiling. Our study provides proof-of-concept for the feasibility of using synthetic Ub-protein conjugates for DUB profiling, providing a basis for deriving rules underlying the removal of ubiquitin from its target proteins, which determines their cellular fates—their ability to interact with ligands, their stability and their subcellular location. It will be interesting see the extent to which these rules also apply to other protein domains whose fold does not resemble that of ubiquitin.

## Methods

### Plasmids

For GOPAL, DIX codon sequences were optimized for expression in *E. coli*. Synthetic sequences (obtained from Integrated DNA Technologies) were inserted as *Nco*I and *Xho*I fragments into pCDF-PylT (encoding *Mb*tRNA_CUA_ with *lpp* promoter and *rrnC* terminator, and spectinomycin resistance marker)^46^. All plasmids were checked by sequencing.

### GOPAL synthesis of DIX-Ub

DIX-Ub conjugates were synthesized by modified GOPAL^19^,^20^.

To express DIX containing site specifically incorporated unnatural amino acid, 50 μl of electro-competent B834(DE3) cells (New England BioLabs) containing pBK-pylRS (kanamycin-resistant plasmid containing *Mb*PylRS *Methanosarcina barkeri* pyrrolysine tRNA synthetase) were transformed with the, spectinomycin-resistant, pCDF-pylT-His6-DIX(XX TAG) plasmid, where XX defines the position of ultimate isopeptide bond formation. SOC medium (250 μl) was then added, and the cells were incubated at 37 °C for 1 h. Luria-Bertani (LB) medium (100 ml) containing spectinomycin (50 μg ml^−1^) and kanamycin (50 μg ml^−1^) was inoculated with the recovered cells (200 μl). After overnight growth, LB medium (1 l) containing spectinomycin (25 μg ml^−1^) and kanamycin (25 μg ml^−1^) was inoculated with the overnight culture (50 ml). Cells were incubated at 37 °C to an OD_600_ of 0.7. **1** (Bachem) dissolved in 1 M sodium hydroxide was added directly to the culture (final concentration 2 mM, with adjustment of pH if necessary). Protein expression was induced after 20 min by adding 0.5 mM isopropyl-β-D-thiogalactopyranoside. Cells were harvested by centrifugation (for 10 min at 12,230*g*) after 6 h at 37 °C, 250 r.p.m.

To purify DIX and its variants, *E. coli* cells (from 1 l cultures) were resuspended in 40 ml of denaturing buffer (50 mM Tris, pH 8, 500 mM NaCl, 6.3 M Gn.HCl, 20 mM imidazole) and sonicated for 2 min. The suspension was clarified by centrifugation at 20 °C (25 min, 20,000*g*). The soluble fraction was incubated with 3 ml Ni-NTA resin (QIAGEN) for 1 h at 20 °C. The resulting slurry was transferred to an empty column and washed with 50 ml buffer, and refolded on beads with sequential dilutions of buffer (20 mM Tris, pH 8, 1 M NaCl, 20 mM imidazole) to remove the Gn.HCl. Protein was then eluted with the same buffer, and proteins were analysed by SDS–polyacrylamide gel electrophoresis (diluting pooled fractions with 20 mM Tris, pH 8, to reduce the NaCl to ∼20 mM). The His-tag was removed with TEV protease (at a ratio of 1:80 TEV/protein, 2 mM dithiothreitol (DTT)) overnight at 4 °C. The protein mixture was purified by ion exchange chromatography (HiTrap Q HP 5 ml) with buffer A (20 mM Tris pH 8, 10 mM NaCl) and buffer B (20 mM pH 8, 1 M NaCl). Pooled fractions were purified further with gel filtration (HiLoad 16/60 Superdex 200 prep grade) in 20 mM Tris, pH 7.4, 100 mM NaCl. Pure protein (DIX**1**_54_ or DIX**1**_58_) was dialysed extensively against 10 mM NH_4_CO_3_ (pH 7.2) with 3 kDa MWCO (molecular weight cut off) membranes (Spectrum Labs) and freeze dried.

Ten micrograms of DIX**1**_54_ or DIX**1**_58_ was dissolved in 530 μl of anhydrous dimethylsulphoxide (DMSO) for Alloc protection (with sonication in a waterbath for 5 min). To this solution, 20.5 μl of di-isopropylethylamine (19 eq per amine) and 88 μl of freshly made 40 mg ml^−1^ Alloc-Osu solution (2.85 eq per amine) were added. The reaction was allowed to proceed in a heating block (25 °C, 400 r.p.m.) for >1 h. The protein was then precipitated three times with ice-cold ether (using 2 ml of ether per 100 μl of protein solution) by vortexing for 15 s and centrifuging for 10 min at 4 °C. The resulting white pellet was air dried for 20 min. To remove the Boc group from the lysine at position 54 or 58 of DIX, the pellet was dissolved in 3:2 TFA/dH_2_O (to 1 mg per 100 μl protein), aided by sonication on ice for 5 min. The protein was deprotected for ∼5 h at 4 °C, and recovered after completion as described above for after protection. Complete protection and deprotection were monitored by ESI-MS (see below).

To create the isopeptide bond between DIX and ubiquitin, Alloc-protected DIX (∼10 mg) and UbSR (8 mg) were dissolved separately in anhydrous DMSO (total 400 μl), sonicated and mixed together. After redissolving, 15 μl of *N*,*N* diisopropylethylamine (DIEA) (100 eq per DIX), 3.6 μl of fresh H-Osu solution (390 mg ml^−1^, 10 eq per UbSR) and 9.4 μl of AgNO_3_ solution (57 mg ml^−1^, 5 eq per UbSR) were added, and incubated at 25 °C in the dark for >16 h. After completion, the protein was recovered as described above for protection and deprotection. The pellet was slightly yellow.

To remove all remaining Alloc-protecting groups, proteins were dissolved in 2:1 DMSO/dH_2_O solution and sonicated for 5 min, and 525 μl of fresh chloro-pentamethylcyclopentadienyl-cyclooctadiene-ruthenium(II) ([Cp*Ru(cod)Cl]) in DMSO (9.5 mg ml^−1^, 1 eq per Alloc group) and 134 μl thiophenol (100 eq per Alloc group) were added, and the reaction mixture was incubated at 50 °C for 2 h. The resulting dark orange solution was precipitated with ice-cold ether, as described above, and the top organic layer was removed gently after each centrifugation.

Following deprotection, the ligated protein was mixed with 10 ml denaturing buffer (20 mM Na_2_HPO_4_ (pH 7.4), 100 mM NaCl, 6 M Gn.HCl) and purified by gel filtration (HiLoad 16/60 Superdex 200 prep grade). Fractions containing DIX-Ub were pooled, dialysed against folding buffer (20 mM Na_2_HPO_4_ (pH 7.4), 100 mM NaCl) with 3 kDa MWCO membranes, and re-purified by gel filtration. Pooled fractions were concentrated with an Amicon Ultra-15 3-kDa MWCO centrifugal filter device (Millipore) and flash-frozen for storage at −80 °C.

### ESI-MS analysis

ESI-MS was carried out using an Agilent 1200 LC-MS system with a 6130 Quadrupole spectrometer. The solvent system consisted of 0.2% (v/v) formic acid in H_2_O as buffer A, and 0.2% (v/v) formic acid in acetonitrile (MeCN) as buffer B. Protein ultraviolet absorbance was monitored at 214 and 280 nm. Protein MS spectra were acquired in positive ionization mode, scanning between 400–2,000 *m*/*z*. Collected spectra were averaged over the entire total ion current. Intact protein masses were calculated via spectral deconvolution using Agilent's LC/MSD Chemstation software with built-in deconvolution tool. The default deconvolution parameters were used (masses between 500 and 50,000 Da, with a maximum allowable charge of +50, a minimum of five peaks in a peak set, a noise cutoff of 1,000 counts and an abundance cutoff at 10% for selected peaks).

In addition, protein mass spectrometry of final products was carried out with an LCT TOF mass spectrometer (Micromass). Samples were prepared with a C4 Ziptip (Millipore) and infused directly in 50% (v/v) aqueous acetonitrile containing 1% formic acid. Samples were injected at 20 μl min^−1^, and calibration was performed in positive ion mode with horse heart myoglobin. Spectra were collected in positive ionization mode, scanning between 400 and 2,000 *m*/*z*, and were composed of a minimum of 30 scans averaged. Molecular masses were obtained by maximum entropy deconvolution using MassLynx version 4.1 software (Micromass).

### Tryptic MS/MS analysis

Polyacrylamide gel slices (1–2 mm) containing purified proteins were prepared for mass spectrometric analysis by manual *in situ* enzymatic digestion. Briefly, the excised protein gel pieces were placed in a well of a 96-well microtitre plate and destained with 50% (v/v) acetonitrile and 50 mM ammonium bicarbonate, reduced with 10 mM DTT, and alkylated with 55 mM iodoacetamide. After alkylation, proteins were digested with 6 ng μl^−1^ trypsin (Promega) at 37 °C overnight. The resulting peptides were extracted in 2% (v/v) formic acid, 2% (v/v) acetonitrile. The digest was analysed by nano-scale capillary LC-MS/MS using an Ultimate U3000 HPLC (ThermoScientific Dionex) to deliver a flow of ∼300 nl min^−1^. Peptides were trapped by a C18 Acclaim PepMap100 5 μm, 100 μm × 20 mm nanoViper (ThermoScientific Dionex) before separation on a C18 Acclaim PepMap100 3 μm, 75 μm × 250 mm nanoViper (ThermoScientific Dionex), and eluted with an acetonitrile gradient. The analytical column outlet was directly interfaced via a nano-flow electrospray ionization source, with a hybrid dual pressure linear ion trap mass spectrometer (Orbitrap Velos, ThermoScientific). Data-dependent analysis was carried out, using a resolution of 30,000 for the full MS spectrum, followed by 10 MS/MS spectra in the linear ion trap. MS spectra were collected over a *m*/*z* range of 300–2,000. MS/MS scans were collected using threshold energy of 35 for collision-induced dissociation. LC-MS/MS data were then searched against a protein database (UniProt KB) with the Mascot search engine programme (Matrix Science). Database search parameters were set with a precursor tolerance of 5 p.p.m. and a fragment ion mass tolerance of 0.8 Da. Two missed enzyme cleavages were allowed, and variable modifications for oxidized methionine, carbamidomethyl cysteine, pyroglutamic acid, phosphorylated serine, threonine and tyrosine, along with GlyGly and LeuArgGlyGly lysine were included. MS/MS data were validated using the Scaffold programme (Proteome Software Inc.). All data were additionally interrogated manually.

### CD spectra

CD spectra were obtained with a Jasco J-810 spectrapolararimeter in 10 mM phosphate (pH6.7), 150 mM NaF at 20 °C using a 0.1 cm pathlength cell. Spectra were averaged over 10 scans and corrected for buffer baseline.

### Protein purification and X-ray crystallography

Human hDvl2 DIX domain bearing the polymerization-deficient mutant M4 (Y27D) was expressed with an N-terminal 6xHis-tag (followed by a TEV cleavage site) in *E. coli* BL21-CodonPlus(DE3)-RILcells (Stratagene). Cells were grown at 37 °C in LB to OD_600_ of 0.9, and the temperature was dropped to 21 °C before isopropyl-β-D-thiogalactoside induction for 4 h. Cells were pelleted, resuspended in 20 mM Tris (pH 8), 200 mM NaCl, 10 mM imidazole supplemented by one complete EDTA-free protease inhibitor cocktail tablet (Roche), lysed by passing twice through an EmulsiFlex-C5 (Avestin) and spun down at 50,000*g* for 30 min. The supernatant was incubated with Ni-NTA Sepharose resin (Qiagen) followed by multiple washes and imidazole elution. Removal of the 6xHis-tag was done by adding 1 mM DTT and TEV protease (1:80) to the pooled eluates at 4 °C overnight, and the DIX domain was purified by gel filtration (HiLoad 26/60 Superdex 75 pg; Amersham Biosciences) using 20 mM Tris (pH 7.4), 200 mM NaCl and 1 mM TCEP as running buffer. Pooled DIX domain fractions were diluted to 40 mM NaCl and further purified by anion exchange chromatography (MonoQ 16/10; Amersham Biosciences) with a linear NaCl gradient (0–1 M). Pure fractions were pooled and concentrated to 50 mg ml^−1^ with Vivaspin 20 concentrators (Sartorius), followed by centrifugation at 100,000*g* for 15 min before crystallization as described^47^ (initial screen of >1,500 different crystallization conditions in 100 nl drops in a 96-well sitting-drop format). Crystals emerged under multiple conditions after growing for 1 day at 19 °C by the vapour diffusion method. Crystals grown in 40% (w/v) PEG400, 0.1 M Tris (pH 8.5) and 0.2 M LiSO_4_ were flash-cooled in liquid nitrogen. X-ray diffraction data were collected in house on a Rigaku FR-E^+^ SuperBright rotating anode with an R-Axis HTC imaging plate detector, and the data were processed. Structural images were created with PyMol.

### SEC-MALS

Hundred microlitres of DIX samples were resolved on a Superdex-75 HR10/300 analytical gel filtration column (GE Healthcare) at 0.5 ml min^−1^ in 20 mM Tris (pH 7.4), 200 mM NaCl, 0.01% (w/v) NaN_3_ before light scattering (on a Wyatt Heleos II 18 angle light scattering instrument coupled to a Wyatt Optilab rEX online refractive index detector) in a standard SEC-MALS format. Heleos detector 12 was replaced with a Wyatt's QELS detector for dynamic light scattering measurements. Protein concentration was determined from the excess differential refractive index based on 0.186 RI increment for 1 g ml^−1^ protein solution. Concentrations and observed scattered intensities at each point in the chromatograms were used to calculate the radius of gyration and absolute molecular mass from the slope and the intercept of the Debye plot, using Zimm's model as implemented in Wyatt's ASTRA software. Autocorrelation analysis of data from the dynamic light scattering detector was also performed using Wyatt's ASTRA software, and the translational diffusion coefficients determined were used to calculate the hydrodynamic radius using the Stokes-Einstein equation.

### Electron microscopy

Purified DIX domain samples (at 100 μM in 20 mM HEPES, pH 7.4, 200 mM NaCl) were applied to 400-mesh carbon-coated grids (Electron Microscopy Sciences), stained with 2% (w/v) uranylacetate and processed^5^ for analysis on an FEI G^2^ Spirit transmission electron microscope. Images were collected at a nominal magnification of × 15,000 and × 30,000 on a Gatan Orius SC200B CCD camera.

### DUB assays

Purified DUBs were used for all assays^48^. For each assay^19^, 4 μg of DIX-Ub (final concentration 7 μM) or 2 μg of Ubiquitin-His_6_ or diUb (final concentration 4 μM) were used, and 2 μg of DUBs were added, except for Cezanne (0.1 μg) and USP5 (0.2 μg). Staining was carried out with the Silver Stain Plus kit (Bio-Rad).

## Author contributions

M.B. and J.W.C. conceived and supervised the project. J.M. and M.F. expressed DIX for ubiquitination, J.M. made and characterized DIX-Ub conjugates, J.M. performed DUB assays, and electron microscopy. J.M. and C.M.J. performed SEC-MALS, M.F. performed the DIX crystallography. R.E. and A.K. provided DUBs. All authors analysed the data and contributed to writing the paper.

## Additional information

**Accession codes.** Coordinates and structure factors for hDvl2 DIX-Y27D have been deposited with the Protein Data Bank under accession code 4WIP.

**How to cite this article:** Madrzak, J. *et al*. Ubiquitination of the Dishevelled DIX domain blocks its head-to-tail polymerization. *Nat. Commun*. 6:6718 doi: 10.1038/ncomms7718 (2015).

## Supplementary Material

Supplementary Information Supplementary Figures 1-14, Supplementary Table 1, Supplementary Methods and Supplementary References

## Figures and Tables

**Table 1:** Data collection and refinement statistics (molecular replacement) for hDvl2 DIX-Y27D.

*Data collection*	
Space group	P2_1_2_1_2_1_
*Cell dimensions*	
*a, b, c* (Å)	56.30, 84.82, 98.26
*α*, *β*, *γ* (°)	90.0, 90.0, 90.0
Resolution (Å)	64.21–2.69
*R*_merge_ (%)*	14.0 (48.0)
Mean *I*/*σ*(*I*)	1.77
Completeness (%)	99.0
Multiplicity	3.1
Complexes in AU	3
	
*Refinement*	
Resolution (Å)	42.51–2.69
No. of reflections	13,481
*R*_work_/*R*_free_ (%)	22.8/24.8
No. of atoms	
Protein	1,994
Ligands	48
Water	13
*B*-factors	
Protein	42.6
Ligands	50.8
Water	34.3
R.m.s. deviations	
Bond length (Å)	0.0124
Bond angle (°)	1.59

^*^Highest resolution shell (in Å) shown in parenthesis.

**Table 2:** Four groups of DUB specificities towards DIX-Ub conjugates.

K58=K54	K54>K58	K54 only	Neither
USP1	USP10	**CYLD**	**USP14**
USP2	USP28	USP25	USP5*
**USP4**	OTUD2	USP27X†	**Trabid**
USP6	OTUD3	Cezanne	A20
USP7	OTUB2	UCHL5	OTUB1
**USP8**	OTUD6A	BAP1	
**USP15**	AMSH-LP	AMSH	
USP16			
USP20			
USP21			
USP36			
USP45			
USP51			
UCHL3			

DUB, deubiquitinase.

Bold, implicated in Wnt signalling.

^*^Indicates residual activity against K54.

^†^Indicates residual activity against K58.
