# Supplementary material for: Ubiquitination of the Dishevelled DIX domain blocks its head-to-tail polymerization
Source: Nat Commun. 2015 Apr 24;6:6718. doi: 10.1038/ncomms7718 (PMC4423210; doi:10.1038/ncomms7718)
Supplement: Supplementary Information — Supplementary Figures 1-14, Supplementary Table 1, Supplementary Methods and Supplementary References [file ncomms7718-s1.pdf]

Ubiquitination of the Dishevelled DIX domain blocks its head-to-tail polymerization

SUPPLEMENTARY INFORMATION

Julia Madrzak[1], Marc Fiedler[1], Christopher M. Johnson[1], Richard Ewan[2], Axel Knebel[2], Mariann Bienz[1,3] and Jason W. Chin[1,3]

[1] MRC Laboratory of Molecular Biology, Cambridge Biomedical Campus, Francis Crick Avenue, Cambridge CB2 0QH, UK

[2] MRC Protein Phosphorylation and Ubiquitylation Unit, College of Life Sciences, University of Dundee, Dow Street, Dundee DD1 5EH, UK

[3] co-corresponding authors

Phone +44 1223 267 088, +44 1223 267 093

Fax +44 1223 268 305

Email chin@mrc-lmb.cam.ac.uk, mb2@mrc-lmb.cam.ac.uk

SUPPLEMENTARY FIGURES

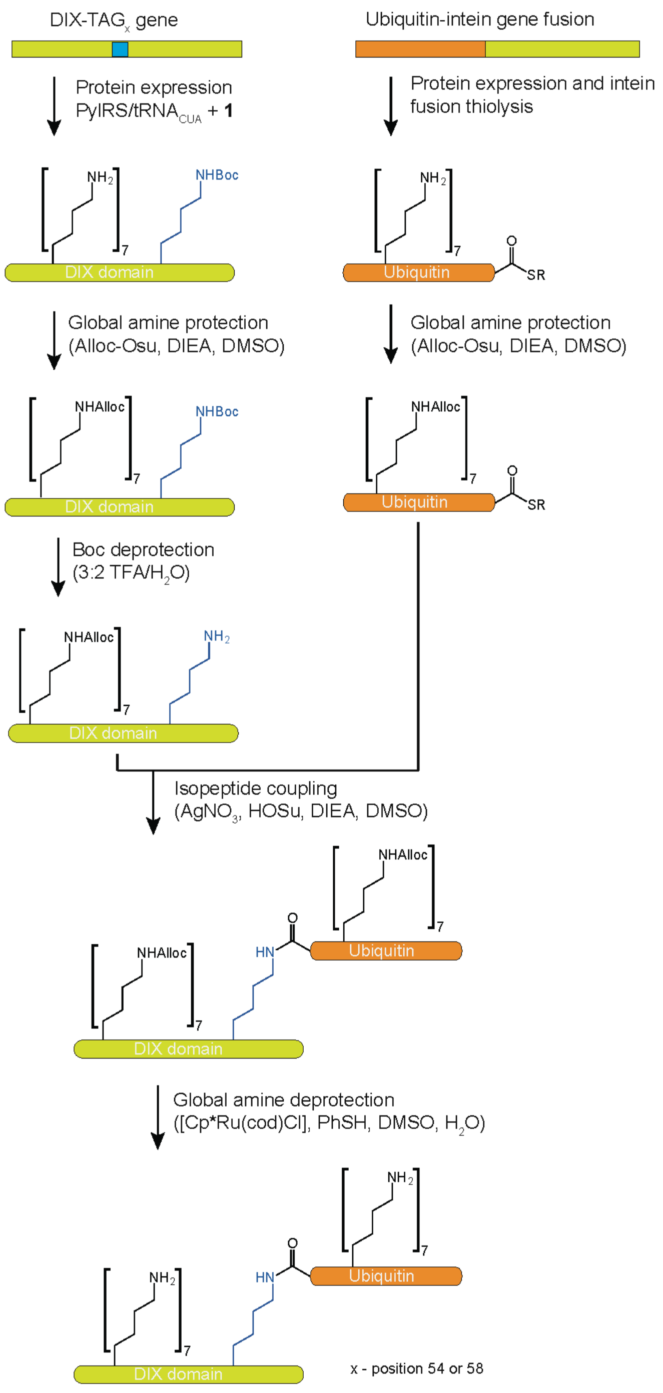

Supplementary Figure 1. Modified GOPAL strategy for site-specific ubiquitination of DIX1.

1 is *Nε*-(*t*-butyloxycarbonyl)-L-lysine; Alloc-Osu, *N*-(allyloxycarbonyl) succinimide; TFA, trifluoroacetic acid; DIEA, *N,N*-diisopropylethylamine; HOSu, *N*-(hydroxy)succinimide;

([Cp*Ru(cod)Cl], chloro-pentamethylcyclopentadienyl-cyclooctadiene-ruthinium(II); PhSH, thiophenol. Cys80 was mutated to serine since Alloc-protecting group are incompatible with cysteine residues.

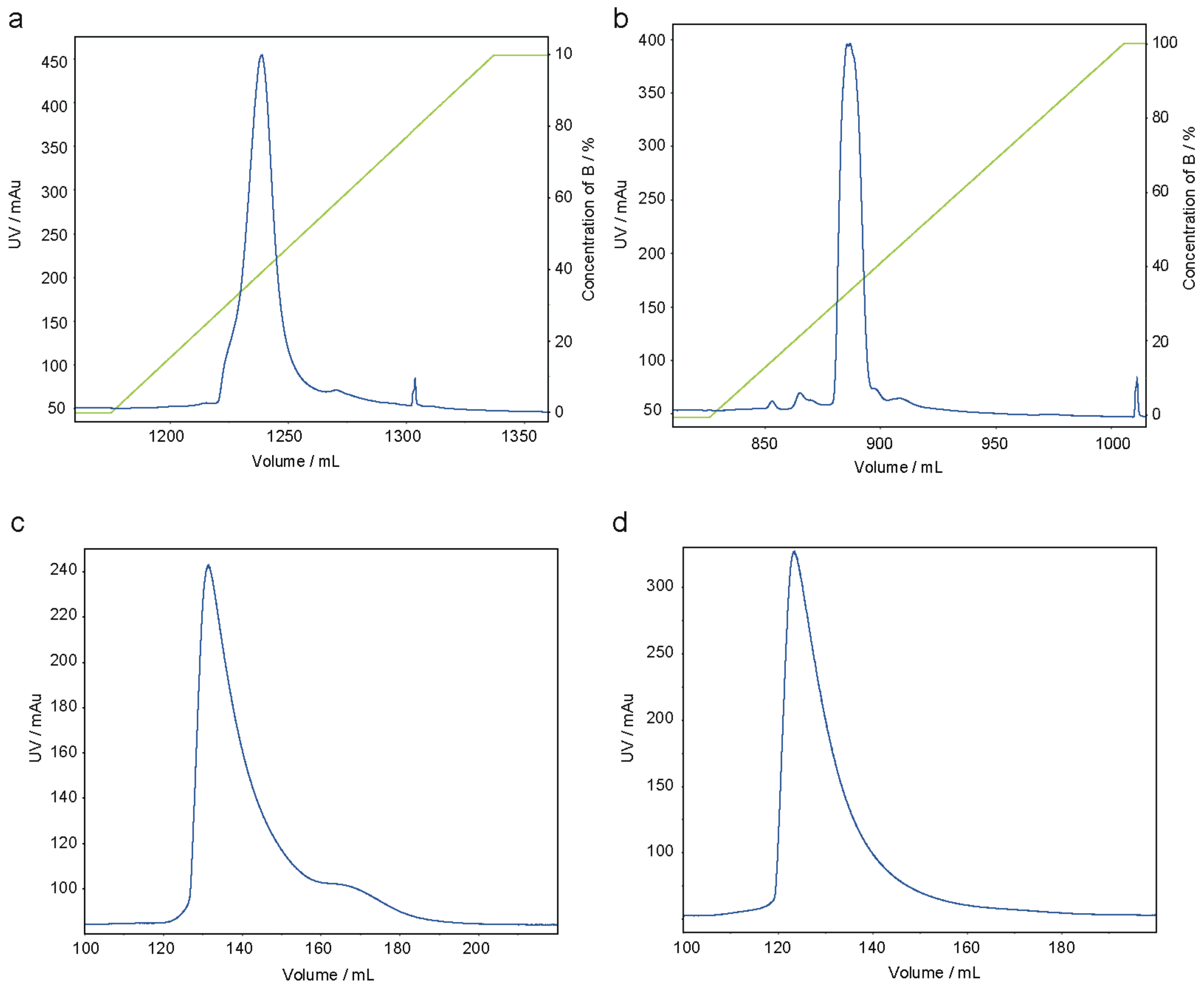

Supplementary Figure 2. Ion exchange and gel filtration chromatography of DIX1$_{54}$ and DIX1$_{58}$. (**a, b**) Elution profiles after ion exchange chromatography (HiTrap Q HP 5 ml) of (**a**) DIX**1**$_{54}$ and (**b**) DIX**1**$_{58}$ after Ni-NTA purification and TEV cleavage. (**c, d**) Elution profiles after gel filtration (HiLoad 16/60 Superdex 200 Prep Grade) of (**c**) DIX**1**$_{54}$ and (**d**) DIX**1**$_{58}$ following purification step in (**a, b**).

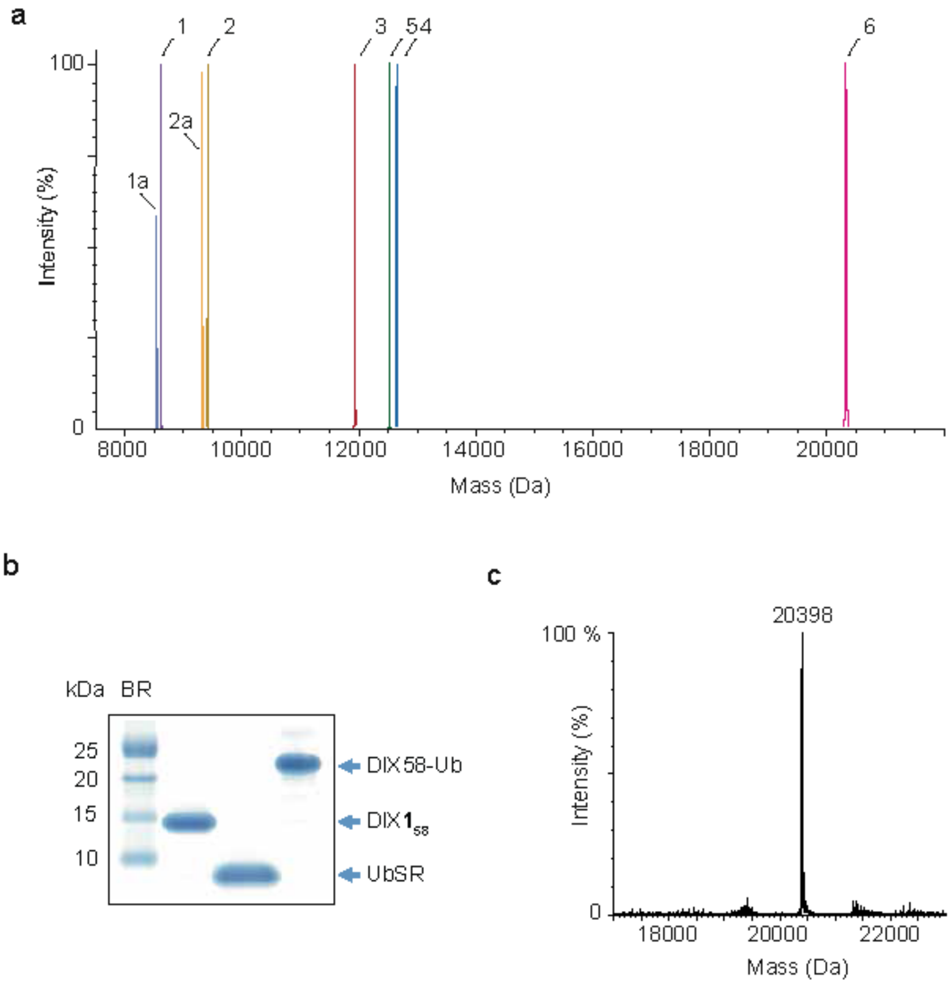

Supplementary Figure 3. Synthesis and characterization of DIX58-Ub.

(**a**) Overlay of LC-MS traces of DIX58-Ub intermediates; violet (1), UbSR, purified ubiquitin-MES thioester; observed mass = 8688, calculated mass = 8689; lilac (1a), hydrolyzed UbCOOH; observed mass = 8564, calculated mass = 8564; ochre (2), UbSR(9Alloc), UbSR after chemical Alloc protection; observed mass = 9445, calculated mass = 9445; yellow (2a), UbCOOH(9Alloc), UbCOOH after chemical Alloc protection; observed mass = 9321, calculated mass = 9320; red (3), DIX**1**$_{58}$, DIX with H-(Boc)-Lys-OH genetically incorporated at K58; observed mass = 11948, calculated mass = 11948; blue (4), DIX**1**$_{58}$(8Alloc), DIX**1**$_{58}$ after chemical protection with Alloc;

observed mass = 12620, calculated mass = 12620; green (5), DIXK_{58}(8Alloc), DIX$\mathbf{1}_{58}$(8Alloc) after Boc deprotection; observed mass = 12520, calculated mass = 12520; pink (6), DIX58-Ub, DIX58-Ub after Alloc deprotection; observed mass = 20394, calculated mass = 20395; **1** is *Nε*-(*t*-butyloxycarbonyl)-L-lysine (H-(Boc)-Lys-OH). These are deconvoluted spectra, all spectra are shown prior to deconvolution in **Supplementary Figures 6-11**. (**b**) SDS-PAGE of starting materials and products for DIX58-Ub synthesis. (**c**) ESI-MS analysis of DIX58-Ub; observed mass = 20398, expected mass = 20395.

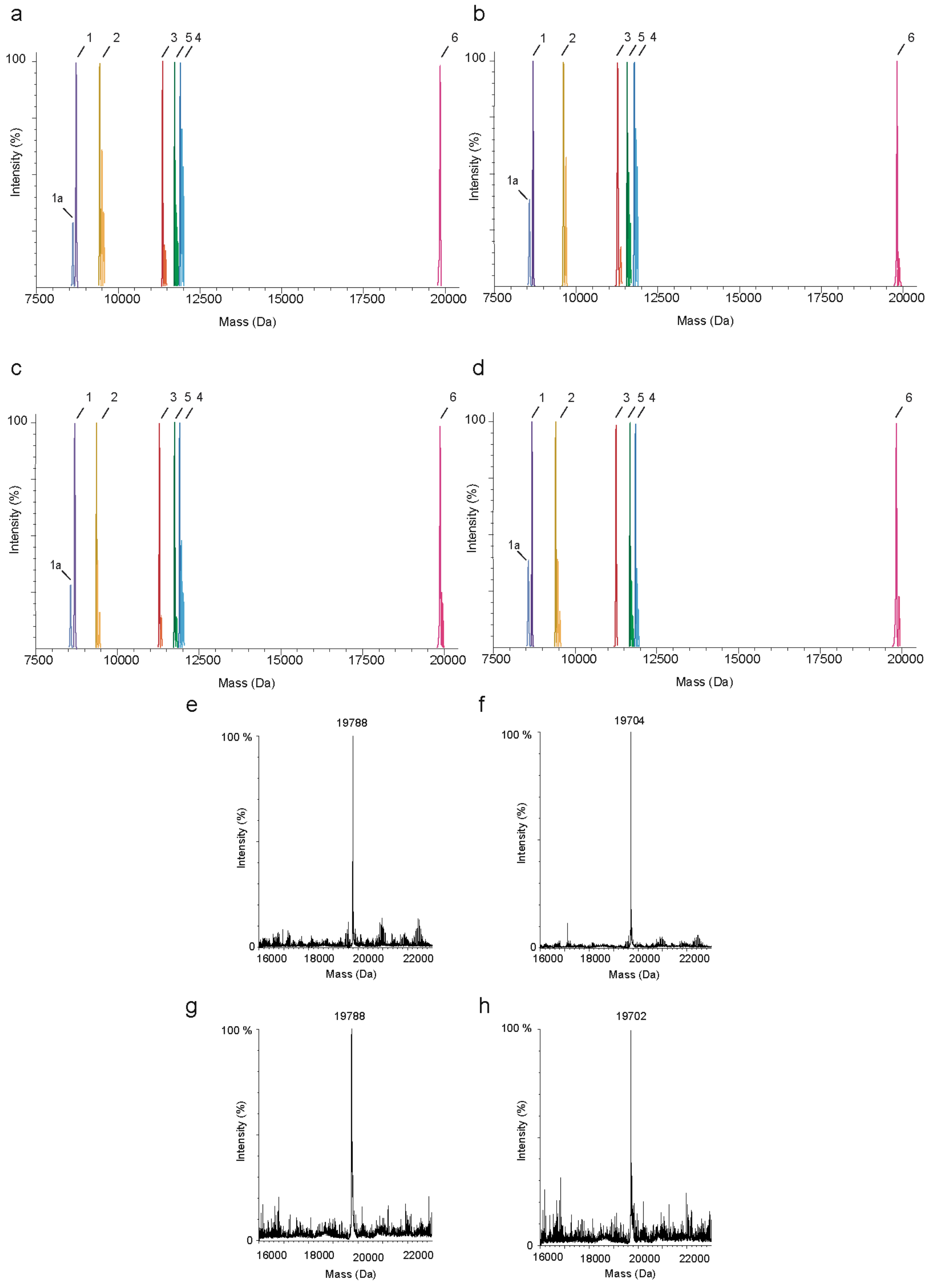
a
b
c
d
1a
1
2
3
5
4
6
100
Intensity (%)
Mass (Da)
7500
10000
12500
15000
17500
20000
e
19788
f
19704
g
19788
h
19702
100 %
0
16000
18000
20000
22000

Supplementary Figure 4. Synthesis and characterization of DIX-Ub* conjugates. (**a**) Overlay of LC-MS traces during synthesis of DIX54-Ub*; violet (1), UbSR, purified ubiquitin-MES thioester; observed mass = 8689, calculated mass = 8689; lilac (1a), hydrolyzed UbCOOH; observed mass = 8564, calculated mass = 8564; yellow (2), UbSR(9Alloc), UbSR after chemical Alloc protection, observed mass = 9445; calculated mass = 9445; red (3), 54 DIX$\mathbf{1}_{54}$, DIX with H-(Boc)-Lys-OH genetically incorporated at position 54; observed mass = 11339, calculated mass = 11339; blue (4), DIX$\mathbf{1}_{54}$(7Alloc), DIX$\mathbf{1}_{54}$ after chemical protection with Alloc; observed mass = 11927, calculated mass = 11927; green (5), DIXK$_{54}$(7Alloc), DIX$\mathbf{1}_{54}$(7Alloc) after Boc deprotection; observed mass = 11827, calculated mass = 11827; pink (6), DIX54-Ub*, DIX site-specifically ubiquitinated at position 54 after Alloc deprotection; observed mass = 19785, calculated mass = 19786. These are deconvoluted spectra, all spectra are shown prior to deconvolution in **Supplementary Figures 6-11.** (**b**) LC-MS traces during synthesis of M2DIX54-Ub*, as in (**a**), except for molecular masses in red (observed mass = 11254, calculated mass = 11254), blue (observed mass = 11758, calculated mass = 11758), green (observed mass = 11656, calculated mass = 11658) and pink (observed mass = 19698, calculated mass = 19701). These are deconvoluted spectra, all spectra are shown prior to deconvolution in **Supplementary Figures 6-11.**. (**c**) LC-MS traces during synthesis of DIX58-Ub*, as in (**a**). These are deconvoluted spectra, all spectra are shown prior to deconvolution in **Supplementary Figures 6-11**. (**d**) Overlay of LC-MS traces during synthesis of M2DIX58-Ub*, as in (**b**). These are deconvoluted spectra, all spectra are shown prior to deconvolution in **Supplementary Figures 6-11**. (**e**) ESI-MS trace of DIX54-Ub*; observed mass = 19788; expected mass = 19786. (**f**) ESI-MS trace of M2DIX54-Ub*; observed mass = 19704, expected mass = 19701. (**g**) ESI-MS trace of DIX58-Ub*; observed mass = 19788, expected mass = 19786; (**h**) ESI-MS trace of M2DIX58-Ub*; observed mass = 19702, expected mass = 19701.

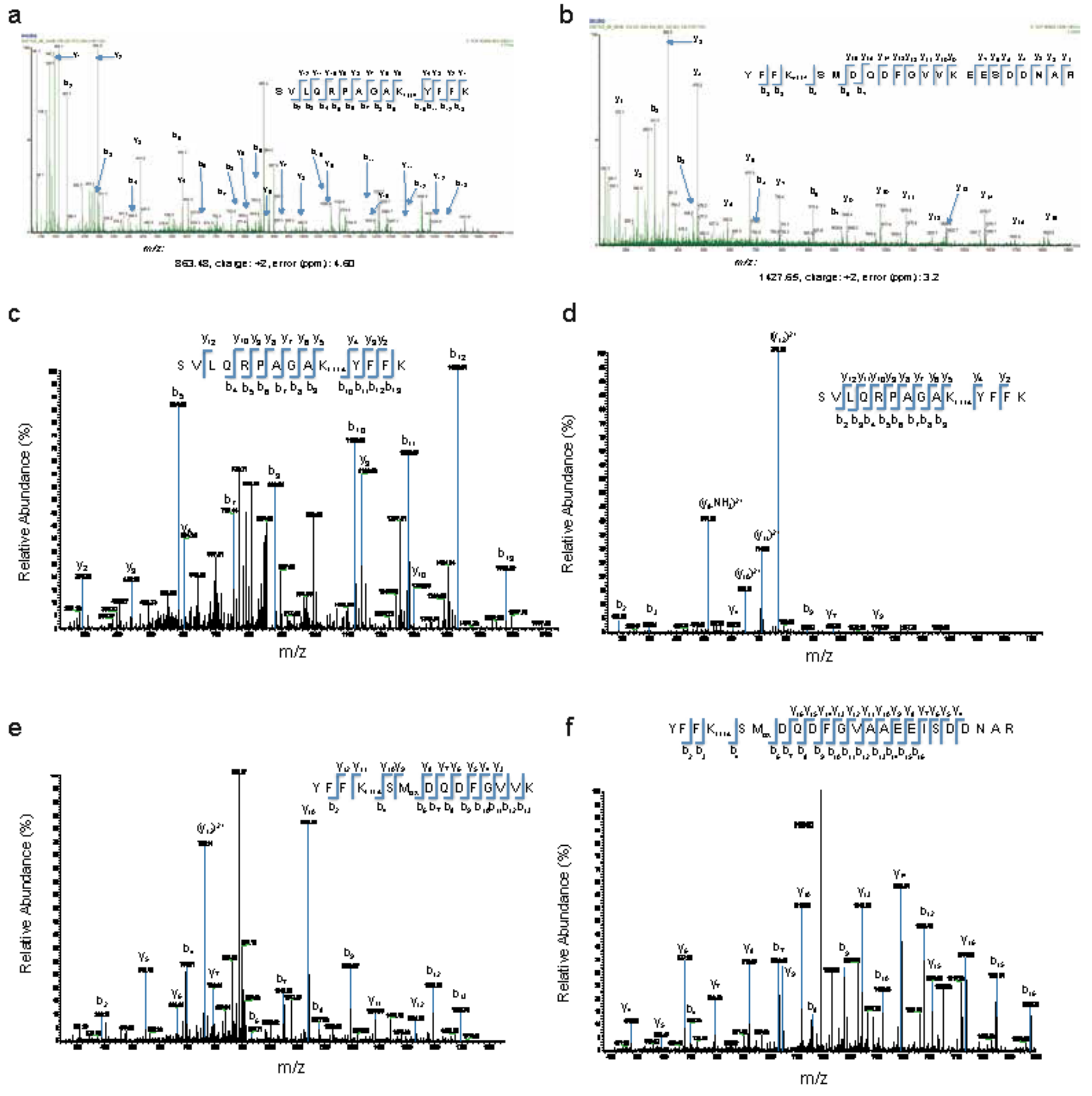

Supplementary Figure 5. Characterization of DIX-Ub conjugates by tryptic MS/MS. Tryptic MS/MS spectra, confirming the expected site of isopeptide bond formation for (**a**) DIX54-Ub, (**b**) DIX58-Ub, (**c**) DIX54-Ub*, (**d**) M2DIX54-Ub*, (**e**) DIX58-Ub* and (**f**) M2DIX58-Ub*.

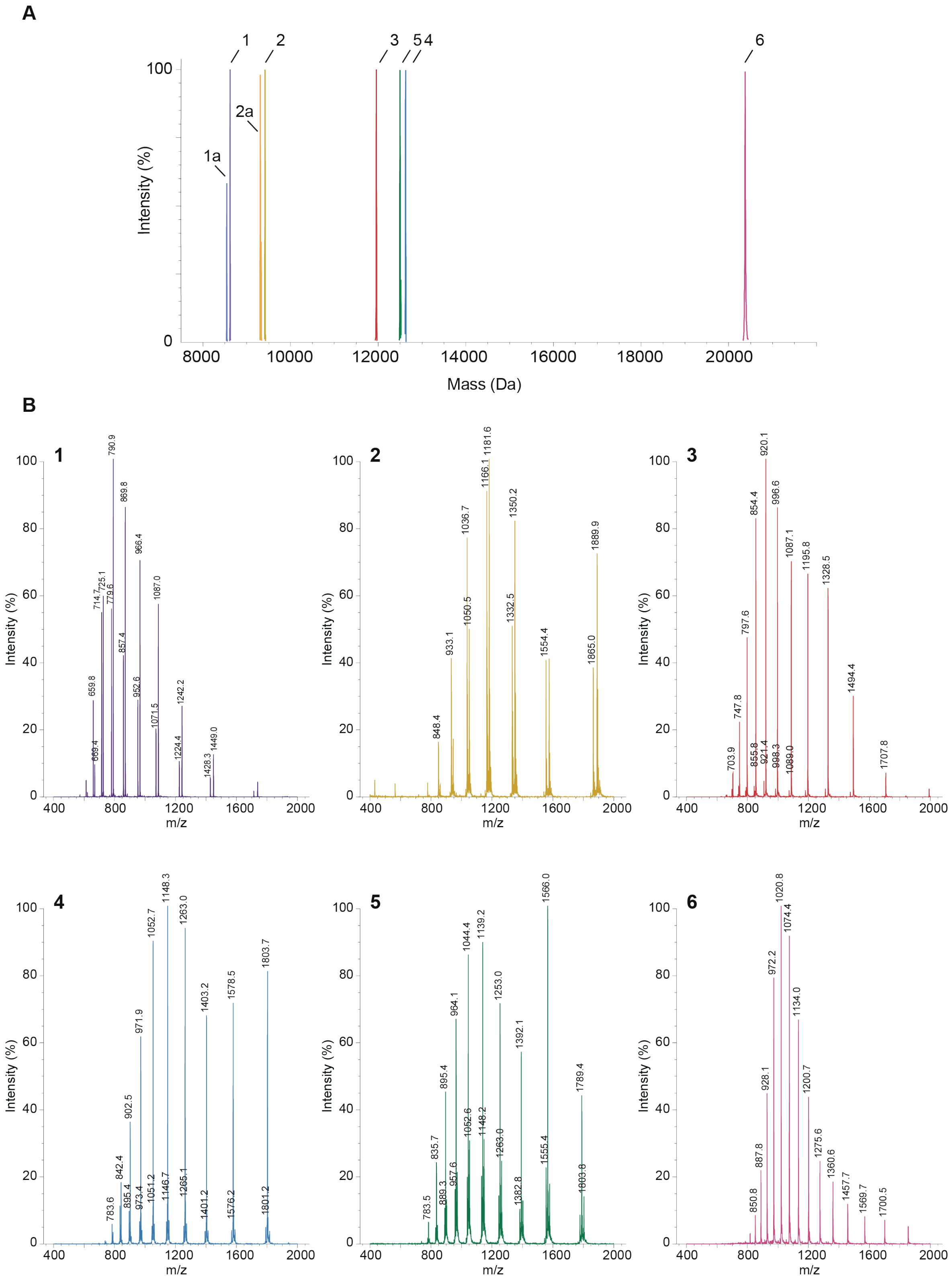

Supplementary Figure 6. The pre-deconvolution spectra of each intermediate in DIX54Ub synthesis. Spectra are colour coded as in Supplementary Figure 3 and 4.

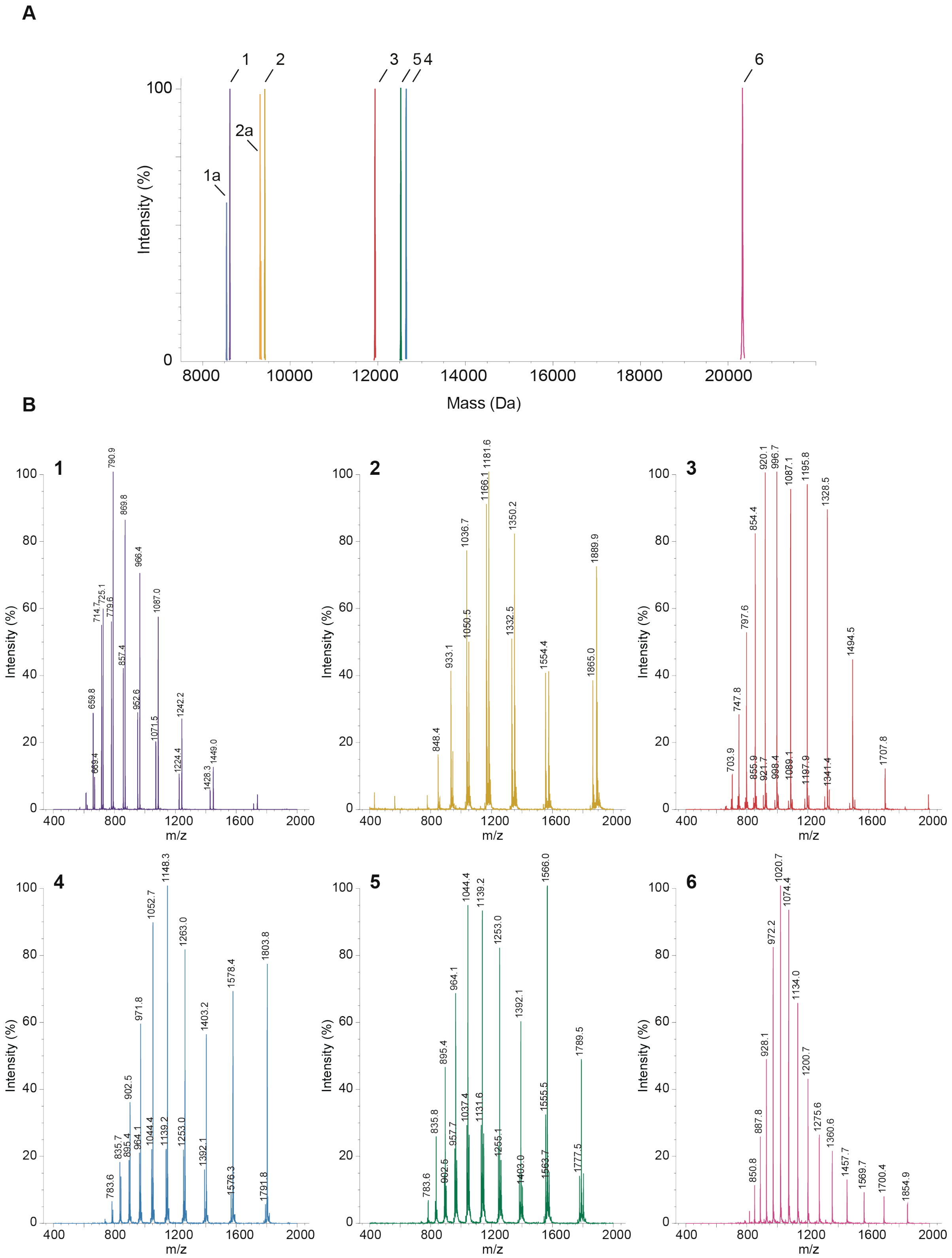

Supplementary Figure 7. The pre-deconvolution spectra of each intermediate in DIX58Ub synthesis. Spectra are colour coded as in Supplementary Figure 3 and 4.

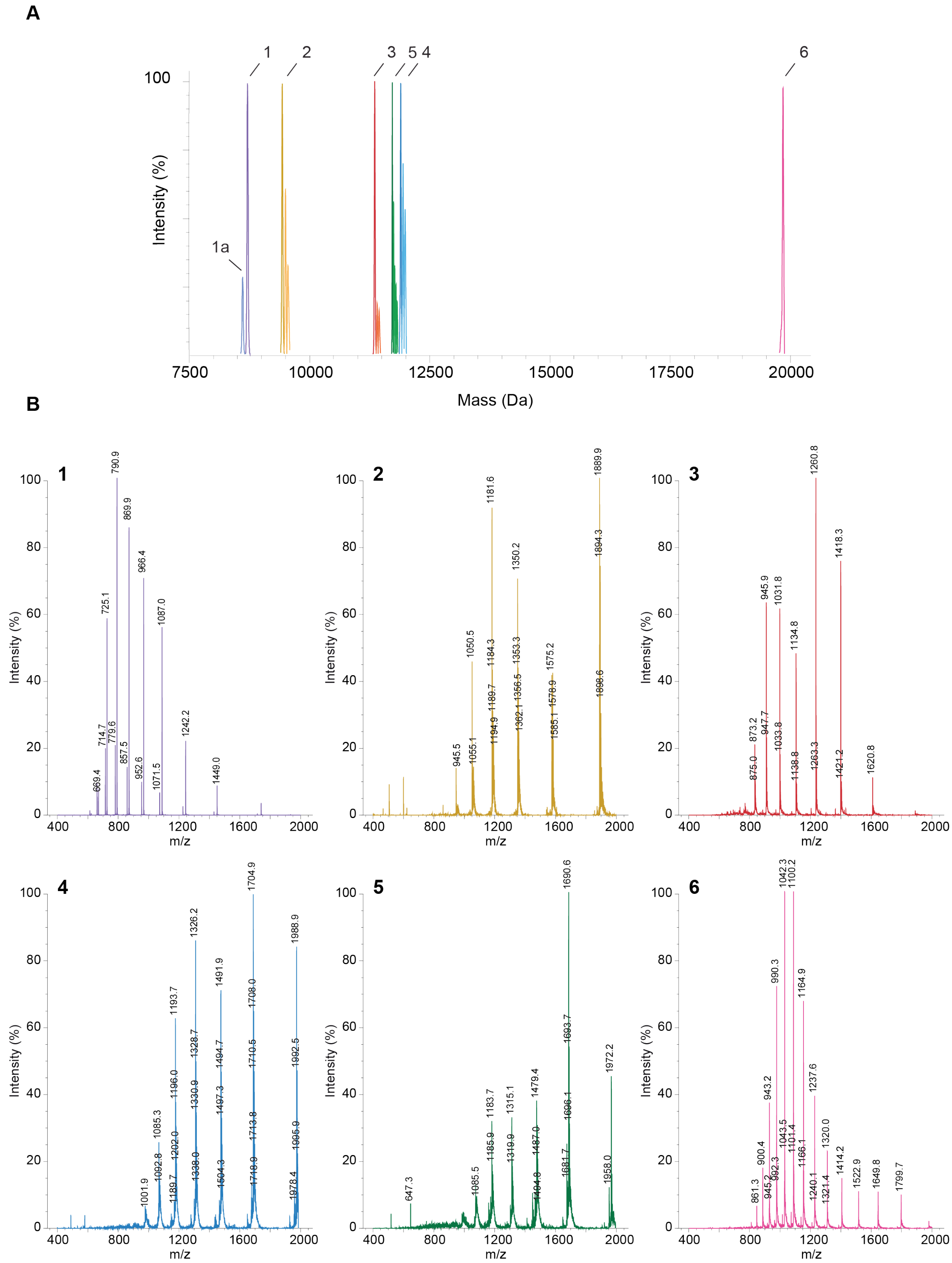

Supplementary Figure 8. The pre-deconvolution spectra of each intermediate in DIX54Ub* synthesis. Spectra are colour coded as in Supplementary Figure 3 and 4.

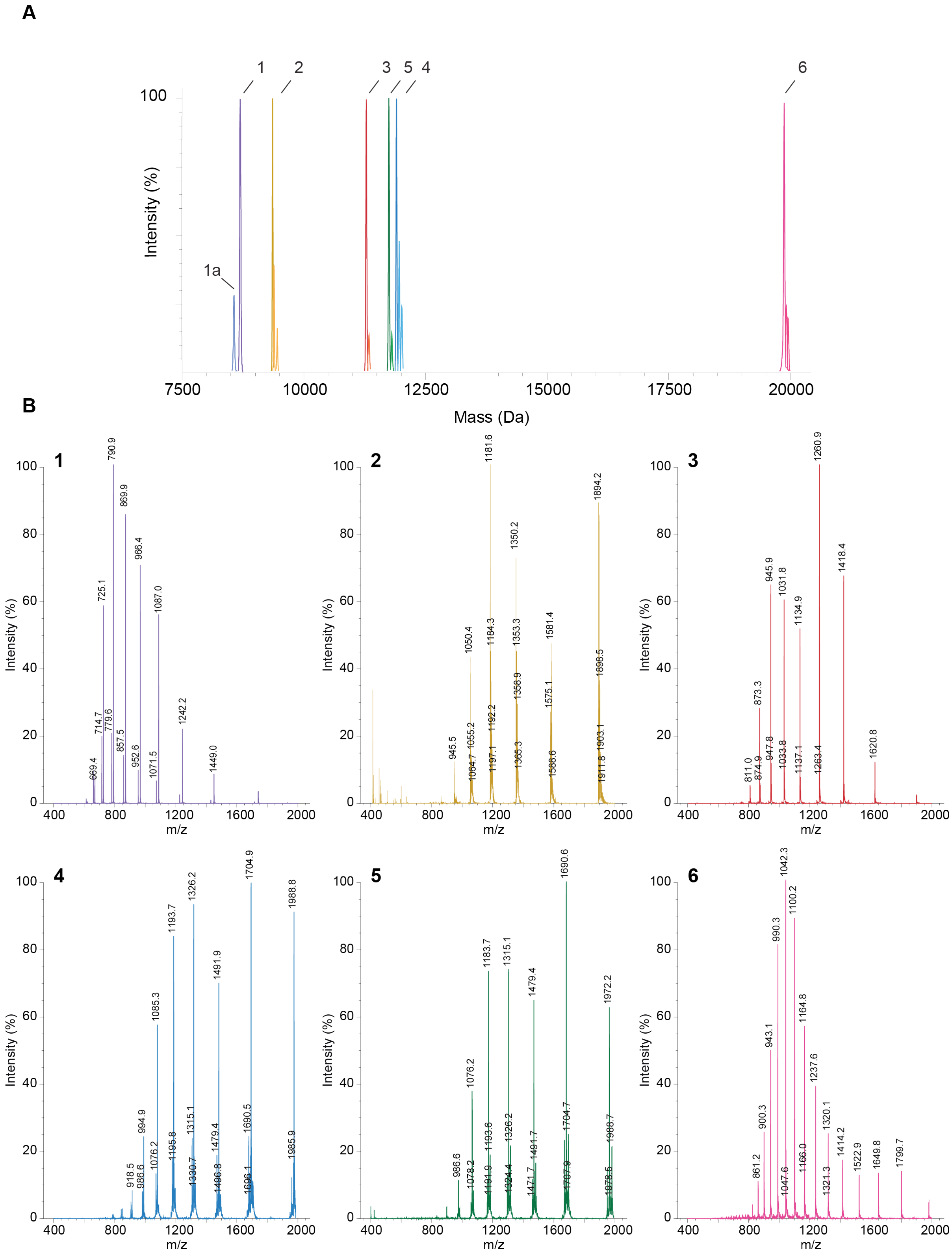

Supplementary Figure 9. The pre-deconvolution spectra of each intermediate in DIX58Ub* synthesis. Spectra are colour coded as in Supplementary Figure 3 and 4.

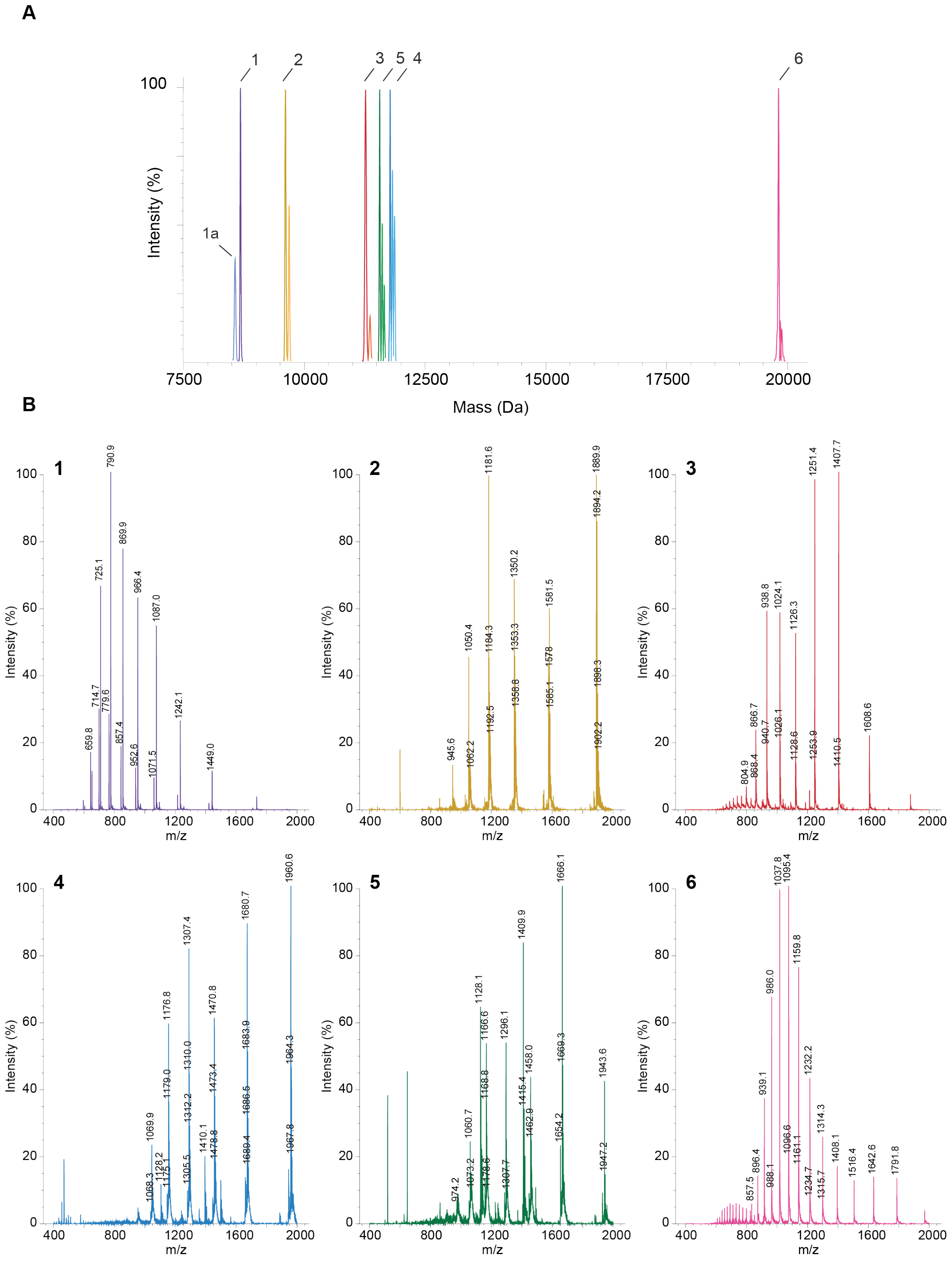

Supplementary Figure 10. The pre-deconvolution spectra of each intermediate in M2DIX54Ub* synthesis. Spectra are colour coded as in Supplementary Figure 3 and 4.

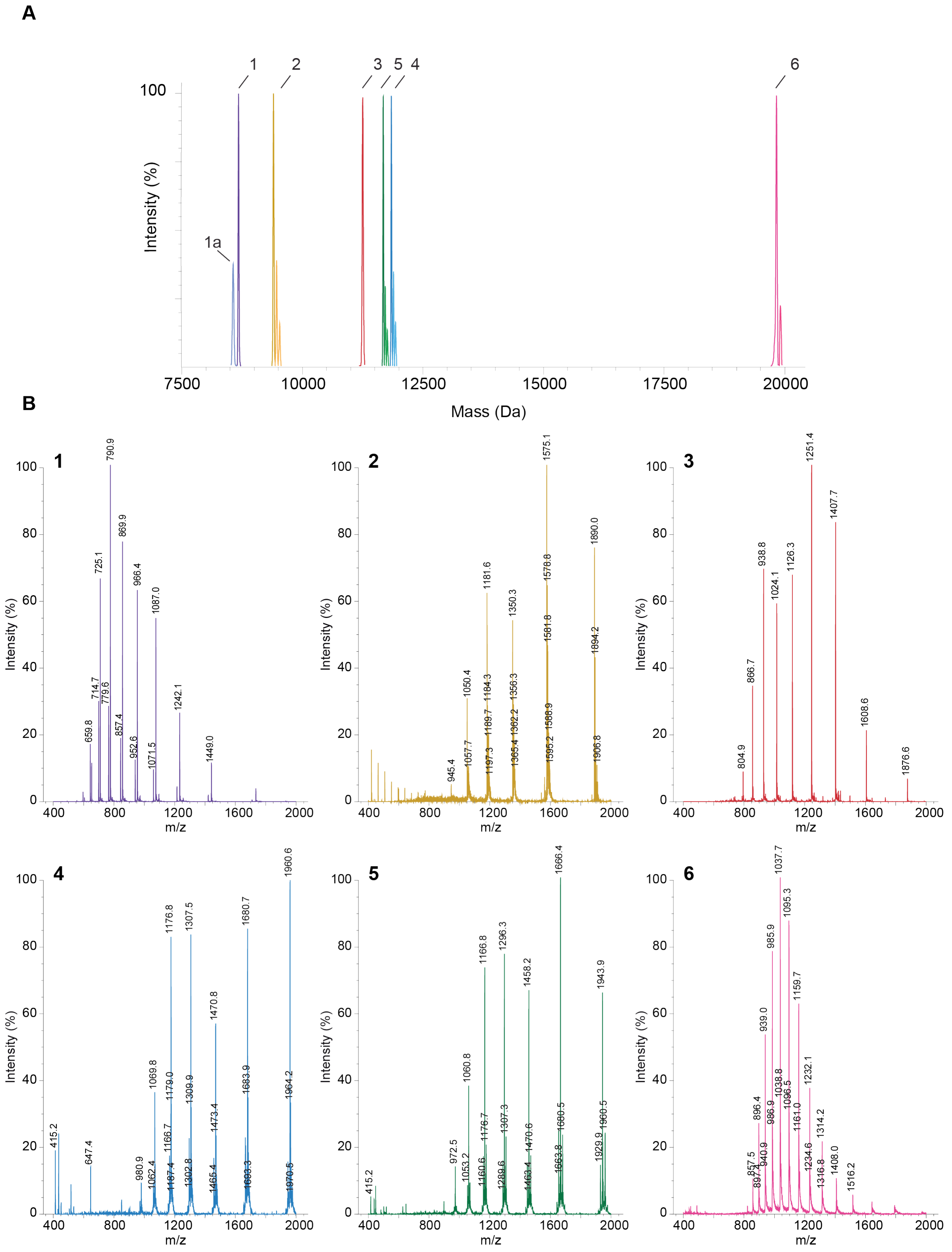

Supplementary Figure 11. The pre-deconvolution spectra of each intermediate in M2DIX58Ub* synthesis. Spectra are colour coded as in Supplementary Figure 3 and 4.

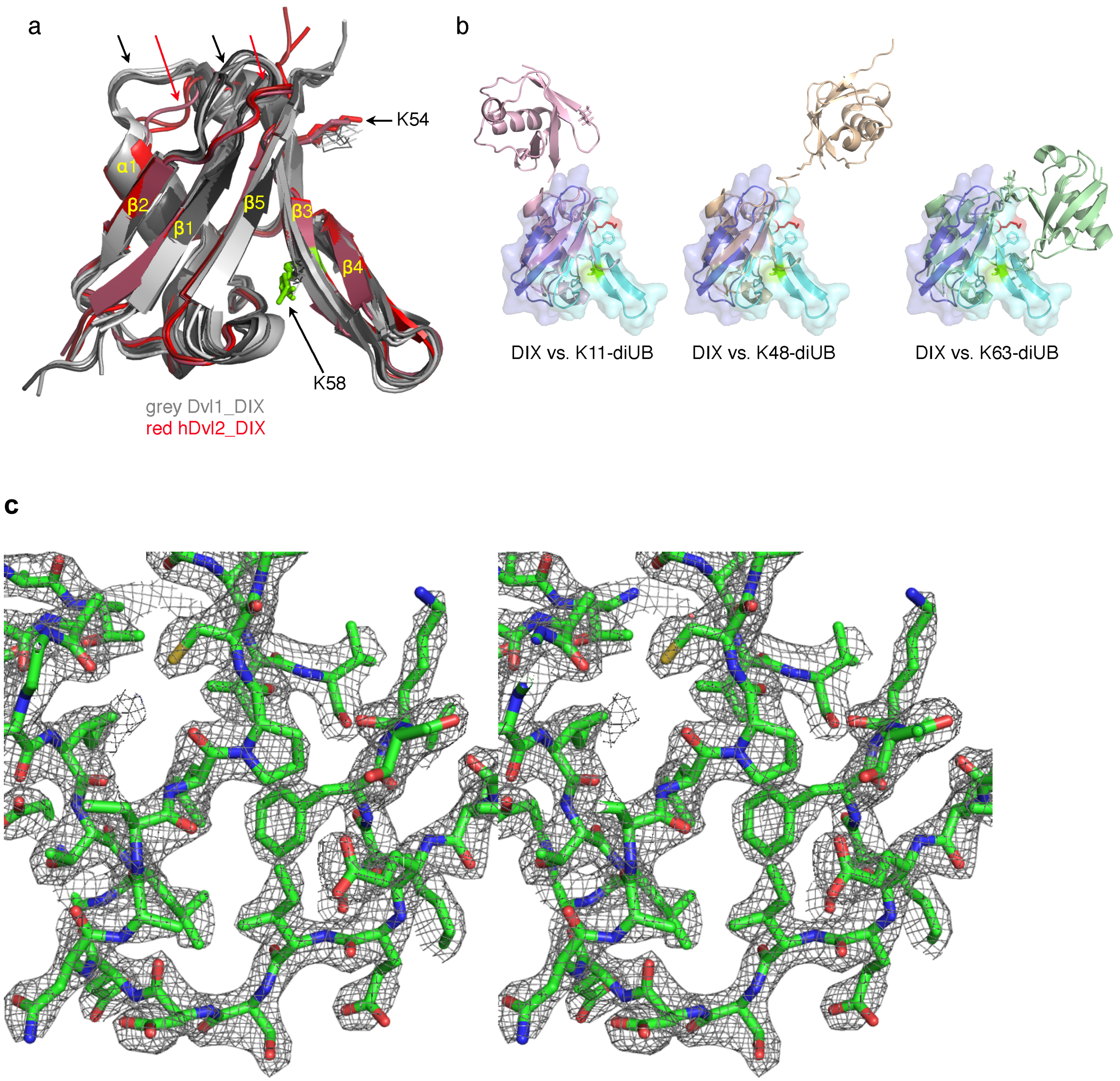

Supplementary Figure 12. Comparison of Dvl DIX structures and overlays with different di-Ubs. (**a**) Ribbon representations of 3 DIX monomers (reds) in the asymmetric unit of the Dvl2 DIX-Y27D crystal (4WIP; rmsd 0.52 Å) superimposed on 8 monomers (greys) found in the Dvl1 DIX-Y17D crystal (3PZ8; rmsd 0.59 Å) [1]; Dvl2 K54 (red) and K58 (green) are shown in stick representation; rmsd values were calculated for the core C_α backbones of all molecules in the asymmetric unit [2]. The main differences between the two domains are found in the loops between β1

and β2, and α1 and β3 (arrows). X-ray diffraction data for the Dvl2 DIX crystal were processed with Mosflm [3] and scaled with Scala [4] (**Table 1**), and the structure was solved by molecular replacement with Phaser [5] based on 3PZ8 [1], and refined at 2.7 Å with Refmac [6]. The models were updated with Coot [7], and analyzed with the CCP4i programs [8]. (**b**) Superimpositions of Dvl2 DIX monomer with K11-diUb [9], K63-diUb [10] and K48-diUb [11]; K54 (red) and K58 (green) are shown as in (**a**), to indicate Ub attachment sites; DIX-K54 is the only, or preferred, DIX-Ub substrate cleaved by K11- and K63-specific DUBs (see main text). (**c**) Stereo view of 2Fo-Fc map at contour level 1.0 σ for the tail region of molecule A of hDvl2 DIX domain; residue numbering indicated for orientation.

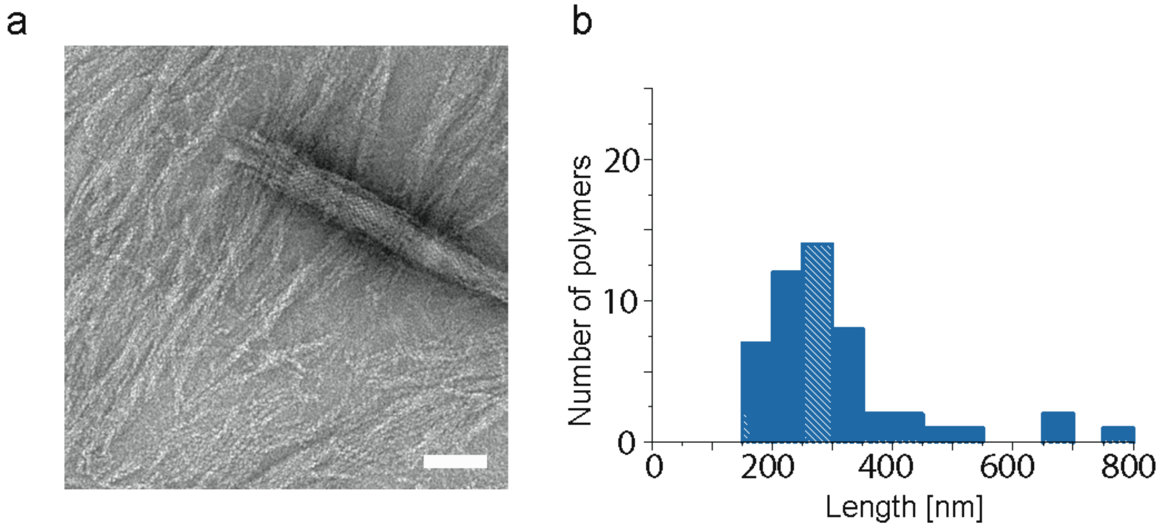

Supplementary Figure 13. Filament and fiber formation by unmodified DIX domain.

(**a**) Transmission EM images of 100 μM unmodified DIX, revealing proto-filaments as well as coalescence of multiple filaments into higher-order fiber; scale bar, 100 nm. (**b**) Length distributions of long DIX filaments; the 5 longest filaments were selected from 10 representative micrographs, and their length was measured (n=50); error bars, standard errors.

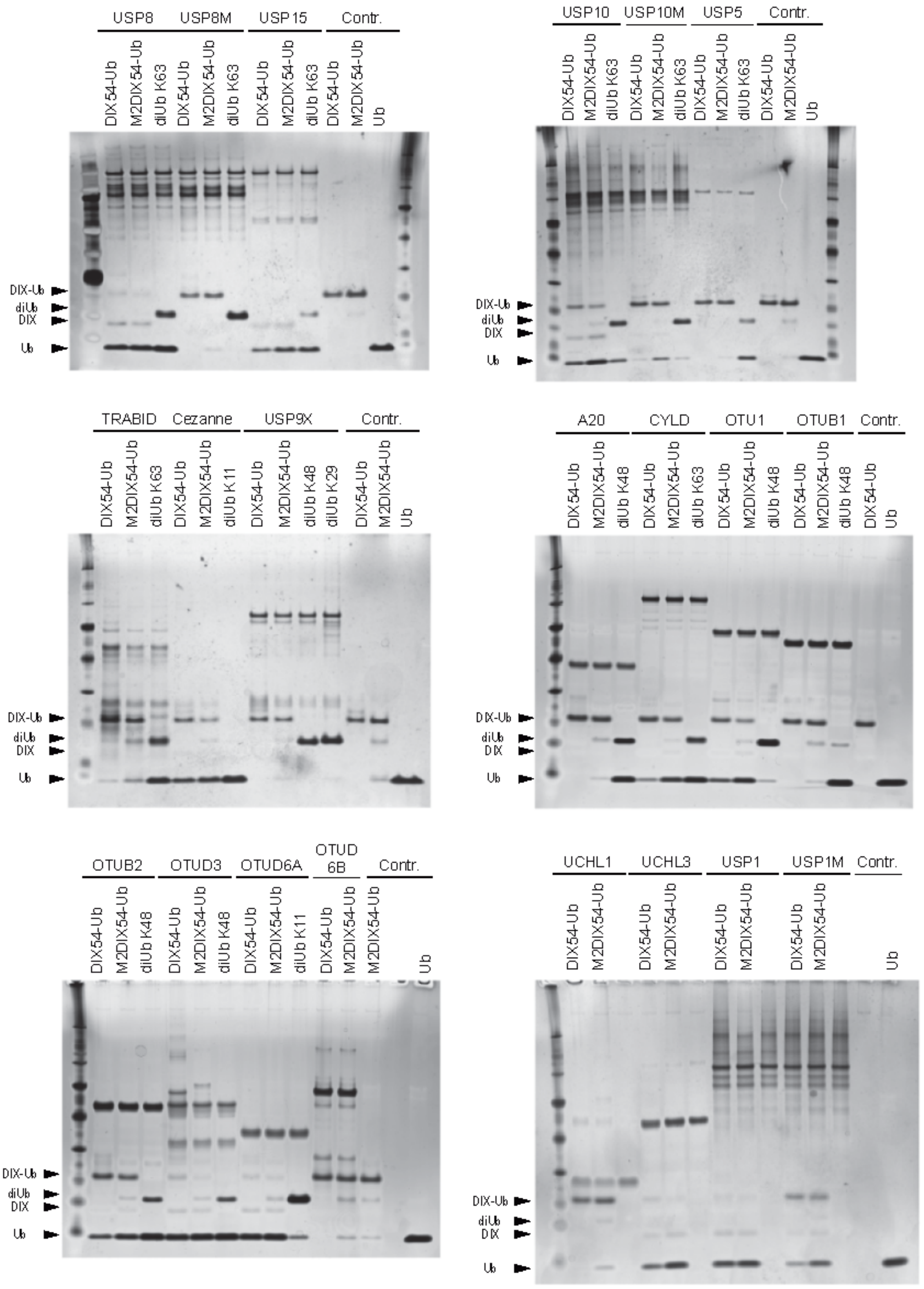
USP8
USP8M
USP15
Contr.
DIX54-Ub
M2DIX54-Ub
diUb K63
DIX54-Ub
M2DIX54-Ub
diUb K63
DIX54-Ub
M2DIX54-Ub
diUb K63
DIX54-Ub
M2DIX54-Ub
Ub
DIX-Ub
diUb
DIX
Ub
USP10
USP10M
USP5
Contr.
DIX54-Ub
M2DIX54-Ub
diUb K63
DIX54-Ub
M2DIX54-Ub
diUb K63
DIX54-Ub
M2DIX54-Ub
diUb K63
DIX54-Ub
M2DIX54-Ub
Ub
DIX-Ub
diUb
DIX
Ub
TRABID
Cezanne
USP9X
Contr.
DIX54-Ub
M2DIX54-Ub
diUb K63
DIX54-Ub
M2DIX54-Ub
diUb K11
DIX54-Ub
M2DIX54-Ub
diUb K48
diUb K29
DIX54-Ub
M2DIX54-Ub
Ub
DIX-Ub
diUb
DIX
Ub
A20
CYLD
OTU1
OTUB1
Contr.
DIX54-Ub
M2DIX54-Ub
diUb K48
DIX54-Ub
M2DIX54-Ub
diUb K63
DIX54-Ub
M2DIX54-Ub
diUb K48
DIX54-Ub
M2DIX54-Ub
diUb K48
DIX54-Ub
Ub
DIX-Ub
diUb
DIX
Ub
OTUB2
OTUD3
OTUD6A
OTUD 6B
Contr.
DIX54-Ub
M2DIX54-Ub
diUb K48
DIX54-Ub
M2DIX54-Ub
diUb K48
DIX54-Ub
M2DIX54-Ub
diUb K11
DIX54-Ub
M2DIX54-Ub
M2DIX54-Ub
Ub
DIX-Ub
diUb
DIX
Ub
UCHL1
UCHL3
USP1
USP1M
Contr.
DIX54-Ub
M2DIX54-Ub
DIX54-Ub
M2DIX54-Ub
DIX54-Ub
M2DIX54-Ub
DIX54-Ub
M2DIX54-Ub
Ub
DIX-Ub
diUb
DIX
Ub

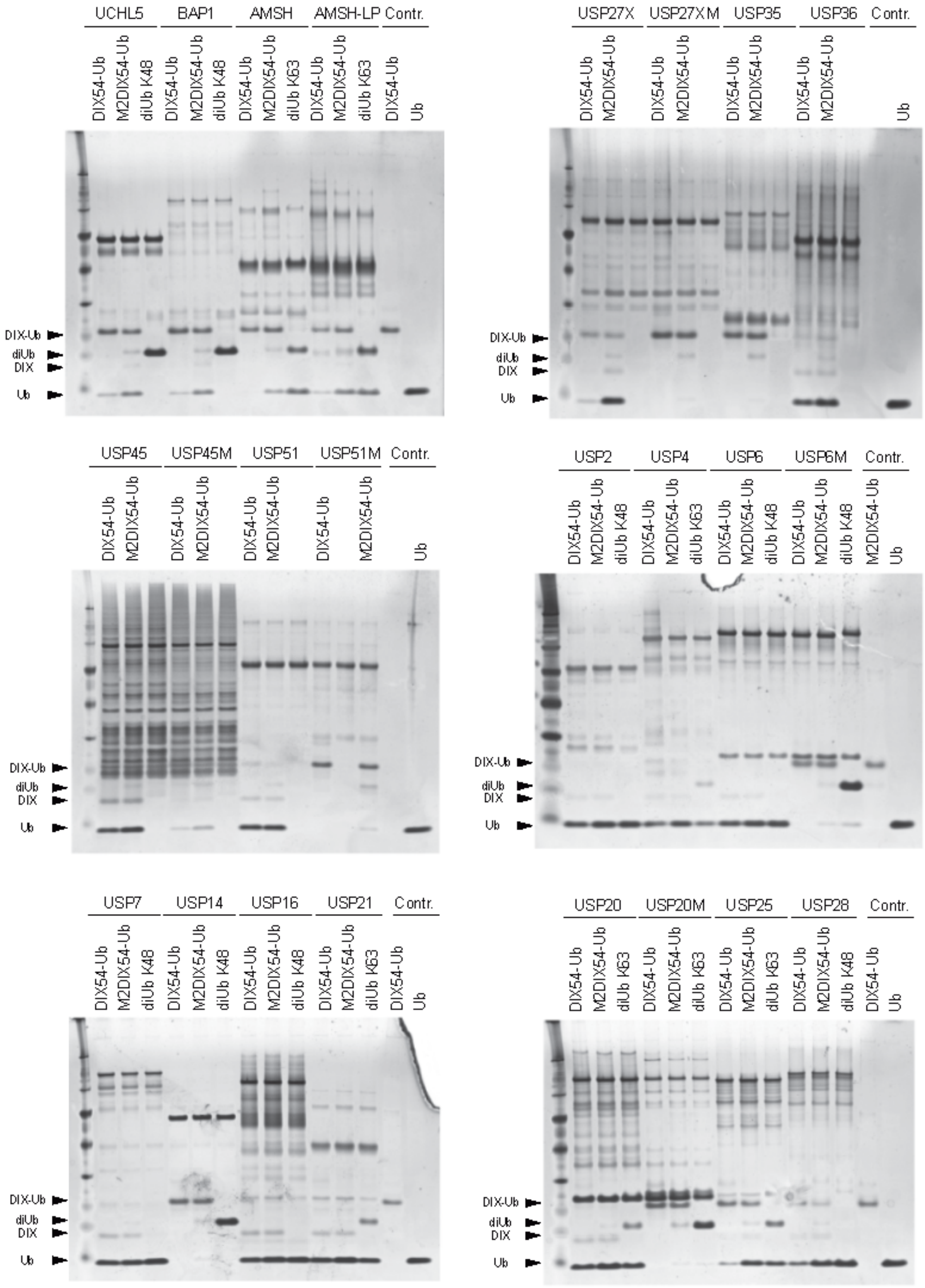
UCHL5 BAP1 AMSH AMSH-LP Contr.
DIX54-Ub M2DIX54-Ub diUb K48 DIX54-Ub M2DIX54-Ub diUb K48 DIX54-Ub M2DIX54-Ub diUb K63 DIX54-Ub M2DIX54-Ub diUb K63 DIX54-Ub Ub
DIX-Ub
diUb
DIX
Ub
USP27X USP27XM USP35 USP36 Contr.
DIX54-Ub M2DIX54-Ub DIX54-Ub M2DIX54-Ub DIX54-Ub M2DIX54-Ub DIX54-Ub M2DIX54-Ub Ub
DIX-Ub
diUb
DIX
Ub
USP45 USP45M USP51 USP51M Contr.
DIX54-Ub M2DIX54-Ub DIX54-Ub M2DIX54-Ub DIX54-Ub M2DIX54-Ub DIX54-Ub M2DIX54-Ub Ub
DIX-Ub
diUb
DIX
Ub
USP2 USP4 USP6 USP6M Contr.
DIX54-Ub M2DIX54-Ub diUb K48 DIX54-Ub M2DIX54-Ub diUb K63 DIX54-Ub M2DIX54-Ub diUb K48 DIX54-Ub M2DIX54-Ub diUb K48 M2DIX54-Ub Ub
DIX-Ub
diUb
DIX
Ub
USP7 USP14 USP16 USP21 Contr.
DIX54-Ub M2DIX54-Ub diUb K48 DIX54-Ub M2DIX54-Ub diUb K48 DIX54-Ub M2DIX54-Ub diUb K48 DIX54-Ub M2DIX54-Ub diUb K63 DIX54-Ub Ub
DIX-Ub
diUb
DIX
Ub
USP20 USP20M USP25 USP28 Contr.
DIX54-Ub M2DIX54-Ub diUb K63 DIX54-Ub M2DIX54-Ub diUb K63 DIX54-Ub M2DIX54-Ub diUb K63 DIX54-Ub M2DIX54-Ub diUb K48 DIX54-Ub Ub
DIX-Ub
diUb
DIX
Ub

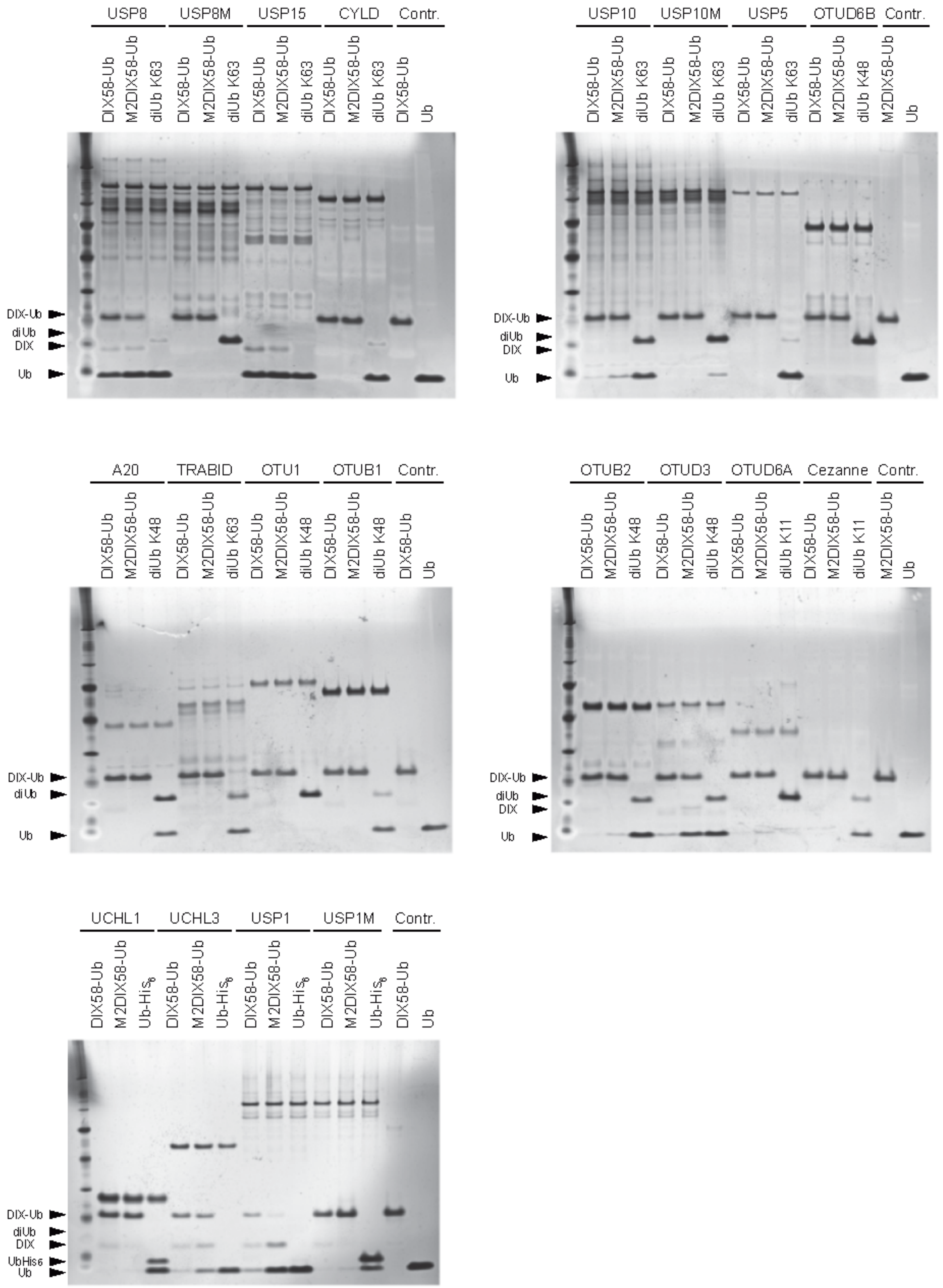
USP8
USP8M
USP15
CYLD
Contr.
DIX58-Ub
M2DIX58-Ub
diUb K63
Ub
DIX-Ub
diUb
DIX
Ub
USP10
USP10M
USP5
OTUD6B
Contr.
diUb K48
A20
TRABID
OTU1
OTUB1
OTUB2
OTUD3
OTUD6A
Cezanne
diUb K11
UCHL1
UCHL3
USP1
USP1M
Ub-His6
UbHis6

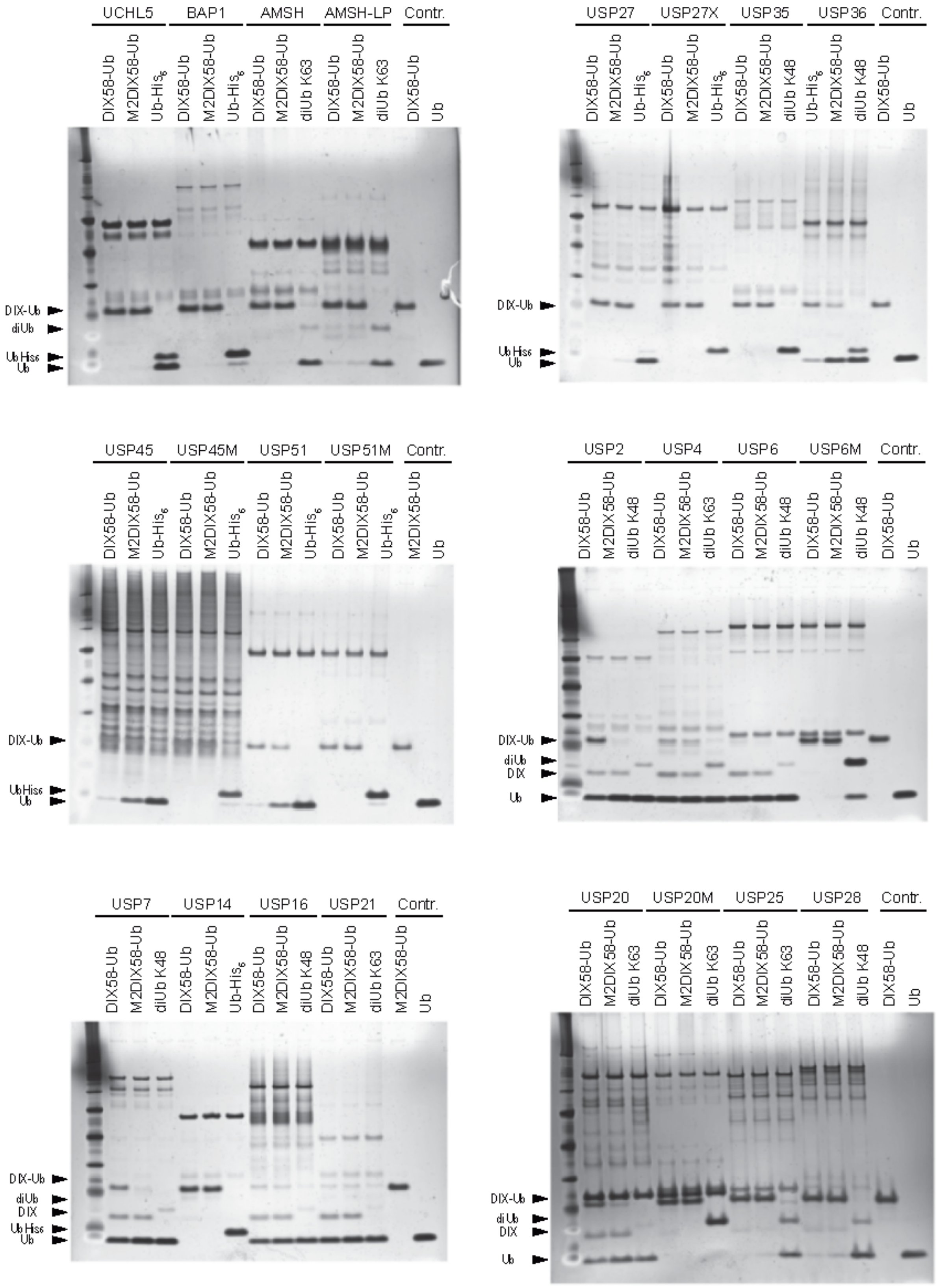
UCHL5
BAP1
AMSH
AMSH-LP
Contr.
DIX58-Ub
M2DIX58-Ub
Ub-His6
diUb K63
Ub
DIX-Ub
diUb
UbHis6
USP27
USP27X
USP35
USP36
diUb K48
USP45
USP45M
USP51
USP51M
USP2
USP4
USP6
USP6M
DIX
USP7
USP14
USP16
USP21
USP20
USP20M
USP25
USP28

Supplementary Figure 14. DUB profiling of DIX-Ub conjugates. Full-length SDS-PAGE of DUB assays shown in main **Fig. 6**, and of additional DUB enzymes as indicated above panels. Note that USP14 proved to be inactive against each substrate, consistent with the dependence of its activity on proteasome association (see main text); however, these *in vitro* DUB assays (without added proteasome) are nevertheless relevant regarding the reported trimming of ubiquitinated Dvl by USP14 since this activity appeared to be proteasome-independent [12].

SUPPLEMENTARY TABLES

Supplementary Table 1. DIX proteins.

Construct	Sequence
DIX	MAHHHHHHSAGENLYFQGAMAGSSTGGGGVGETKVIYHLDEEETPYLVKIPVPAERITLGDFKSVL QRPAGAKYFFKSMDQDFGVVKEEISDDNARLPSFNGRVVSWLVSSDNPQPEMAPPVHEPR
DIX54-Ub	MAHHHHHHSAGENLYFQGAMAGSSTGGGGVGETKVIYHLDEEETPYLVKIPVPAERITLGDFKSVL QRPAGA*YFFKSMDQDFGVVKEEISDDNARLPSFNGRVVSWLVSSDNPQPEMAPPVHEPR
DIX58-Ub	MAHHHHHHSAGENLYFQGAMAGSSTGGGGVGETKVIYHLDEEETPYLVKIPVPAERITLGDFKSVL QRPAGAKYFF*SMDQDFGVVKEEISDDNARLPSFNGRVVSWLVSSDNPQPEMAPPVHEPR
DIX*	MAGSSTGGGGVGETKVIYHLDEEETPYLVKIPVPAERITLGDFKSVLQRPAGAKYFFKSMDQDFGVV KEEISDDNARLPSFNGRVVSWLVSSDNPQPENLYFQGMAPPVHEPRHHHHHH
DIX54-Ub*	MAGSSTGGGGVGETKVIYHLDEEETPYLVKIPVPAERITLGDFKSVLQRPAGA**1**YFFKSMDQDFGVV KEEISDDNARLPSFNGRVVSWLVSSDNPQPENLYFQGMAPPVHEPRHHHHHH
M2DIX54-Ub*	MAGSSTGGGGVGETKVIYHLDEEETPYLVKIPVPAERITLGDFKSVLQRPAGA**1**YFFKSMDQDFGVA AEEISDDNARLPSFNGRVVSWLVSSDNPQPENLYFQGMAPPVHEPRHHHHHH
DIX58-Ub*	MAGSSTGGGGVGETKVIYHLDEEETPYLVKIPVPAERITLGDFKSVLQRPAGAKYFF**1**SMDQDFGVV KEEISDDNARLPSFNGRVVSWLVSSDNPQPENLYFQGMAPPVHEPRHHHHHH
M2DIX58-Ub*	MAGSSTGGGGVGETKVIYHLDEEETPYLVKIPVPAERITLGDFKSVLQRPAGAKYFF**1**SMDQDFGVA AEEISDDNARLPSFNGRVVSWLVSSDNPQPENLYFQGMAPPVHEPRHHHHHH

Protein sequences of the DIX plasmids used for GOPAL (*, constructs used for DUB assays; **1**, *Nε*-{*t-butyloxycarbonyl*}-L-Lysine).

SUPPLEMENTARY METHODS

Protein expression. 50 µl of electro-competent B834(DE3) cells (New England BioLabs) containing pBK-pylRS (kanamycin-resistant plasmid containing constitutive *Mb*PylRS *Methanosarcina barkeri* pyrrolysine tRNA synthetase) were transformed with pCDF-pylT-DIX. SOC medium (250 µl) was then added, and the cells were incubated at 37 °C for 1 h. LB medium (100 ml) containing spectinomycin (50 µg ml^{-1}) and kanamycin (50 µg ml^{-1}) was then inoculated with the recovered cells (200 µl). After overnight growth, LB medium (1 l) containing spectinomycin (25 µg ml^{-1}) and kanamycin (25 µg ml^{-1}) was inoculated with the overnight culture (50 ml). Cells were incubated at 37 °C to an OD_{600} of 0.7. H-(Boc)Lys-OH (Bachem) dissolved in 1 M sodium hydroxide was added directly to the culture (final concentration 2 mM, with adjustment of pH if necessary), and cells were induced after 20 min by adding 0.5 mM isopropyl-β-D-thiogalactopyranoside, and harvested by centrifugation (for 10 min at 12,230 g) after 6 h at 37 °C.

Protein purification. *E. coli* cells (from 1 l cultures) were suspended in 40 ml of denaturing buffer (50 mM Tris pH 8, 500 mM NaCl, 6.3 M Gn.HCl, 20 mM imidazole) and sonicated for 2 min. The suspension was clarified by centrifugation at 20 °C (for 25 min at 20,000 g). The soluble fraction was incubated with 3 ml Ni-NTA resin (QIAGEN) for 1 h at 20 °C. The slurry was then transferred to an empty column and washed with 50 ml buffer, and refolded on beads with sequential dilutions of buffer (20 mM Tris, pH 8, 1 M NaCl, 20 mM imidazole) to remove the Gn.HCl. Protein was then eluted with the same buffer, and proteins were analyzed by SDS-PAGE (diluting pooled fractions with 20 mM Tris pH 8, to reduce the NaCl to ~20 mM). The His-tag was removed with TEV enzyme (at a ratio of 1:80 TEV:protein, 2 mM DTT) overnight at 4 °C, and the protein mixture was purified

by ion exchange chromatography (HiTrap Q HP 5 ml) with buffer A (20 mM Tris pH 8, 10 mM NaCl) and buffer B (20 mM pH 8, 1 M NaCl). Pooled fractions were purified further with gel filtration (HiLoad 16/60 Superdex 200 prep grade) in 20 mM Tris pH 7.4, 100 mM NaCl. Pure protein was dialyzed extensively against 10 mM NH_4CO_3 pH 7.2 with 3 kDa MWCO membranes (Spectrum Labs) and freeze dried.

Alloc protection and Boc deprotection. 10 mg of protein was dissolved in 530 µl of anhydrous DMSO for Alloc protection (with sonication in a waterbath for 5 min). To this solution were added 20.5 µl of di-isopropylethylamine (19 eq/amine) and 88 µL of freshly made 40 mg ml^{-1} Alloc-Osu solution (2.85 eq/amine). The reaction was allowed to proceed in a heating block at 25 °C (400 rpm) for >1 h. After completion, the protein was subjected to three rounds of precipitation with ice-cold ether (using 2 ml of ether per 100 µl of protein solution) by vortexing for 15 s and centrifuging for 10 min at 4 °C, and the white pellet was air dried for 20 min. For deprotection, this pellet was dissolved in 3:2 TFA/dH_2O (to 1 mg 100 μl^{-1} protein), aided by sonication on ice for 5 min. Deprotection was for ~5 h at 4 °C, and the protein was recovered after completion as after protection. Complete protection and deprotection were monitored by ESI-MS (see below).

Ligation. Protected DIX (~10 mg) and UbSR (8 mg) were dissolved separately in anhydrous DMSO (total 400 µl), sonicated and mixed together after re-dissolving. 15 µl of DIEA (100 eq/DIX), 3.6 µl of fresh H-Osu solution (390 mg ml^{-1}, 10 eq/UbSR) and 9.4 µl of $AgNO_3$ solution (57 mg ml^{-1}, 5 eq/UbSR) were added, and incubated at 25 °C in the dark for >16 h. After completion, the protein was recovered as described above for protection and deprotection. The pellet was slightly yellow.

Global Alloc deprotection. Proteins were dissolved in 2:1 DMSO:dH_2O solution and sonicated for 5 min, and 525 µl of fresh chloro-pentamethylcyclopentadienyl-cyclooctadiene-ruthenium(II) ([Cp*Ru(cod)Cl]) in DMSO (9.5 mg ml^{-1}, 1eq/Alloc group) and 134 µl thiophenol (100 eq/Alloc group) were added, and the reaction mixture was incubated at 50 °C for 2 h. The resulting dark orange solution was precipitated with ice-cold ether, as described above, and the top organic layer was removed gently after each centrifugation.

Purification and renaturation of ligation products. Ligated protein was mixed with 10 ml denaturing buffer (20mM Na_2HPO_4 pH 7.4, 100 mM NaCl, 6 M Gn.HCl) and purified by gel filtration (HiLoad 16/60 Superdex 200 prep grade). Fractions containing DIX-Ub were pooled, dialyzed against folding buffer (20 mM Na_2HPO_4 pH 7.4, 100 mM NaCl) with 3 kDa MWCO membranes, and re-purified by gel filtration. Pooled fractions were concentrated with an Amicon Ultra-15 3 kDa MWCO centrifugal filter device (Millipore) and flash-frozen for storage at -80 °C.

ESI-MS analysis. ESI-MS was carried out using an Agilent 1200 LC-MS system with a 6130 Quadrupole spectrometer. The solvent system consisted of 0.2 % (v/v) formic acid in H_2O as buffer A, and 0.2 % (v/v) formic acid in acetonitrile (MeCN) as buffer B. Protein UV absorbance was monitored at 214 and 280 nm. Protein MS spectra were acquired in positive ionisation mode, scanning between 400-2000 m/z. Collected spectra were averaged over the entire total ion current (TIC). Intact protein masses were calculated via spectral deconvolution using Agilent's LC/MSD Chemstation software with built-in deconvolution tool. The default deconvolution parameters were used (masses between 500-50000 Da, with a maximum allowable charge of +50, a minimum of 5 peaks in a peak set, a noise cut off of 1000 counts and an abundance cut off at 10% for selected peaks).

Additionally, protein mass spectrometry of final products was carried out with an LCT TOF mass spectrometer (Micromass). Samples were prepared with a C4 Ziptip (Millipore) and infused directly in 50 % (v/v) aqueous acetonitrile containing 1 % formic acid. Samples were injected at 20 µl min^{-1}, and calibration was performed in positive ion mode with horse heart myoglobin. Spectra were collected in positive ionisation mode, scanning between 400-2000 m/z, and were composed of a minimum of 30 scans averaged. Molecular masses were obtained by maximum entropy deconvolution using MassLynx version 4.1 software (Micromass).

Tryptic MS/MS analysis. Polyacrylamide gel slices (1-2 mm) containing purified proteins were prepared for mass spectrometric analysis by manual in situ enzymatic digestion. Briefly, the excised protein gel pieces were placed in a well of a 96-well microtitre plate and destained with 50% (v/v) acetonitrile and 50 mM ammonium bicarbonate, reduced with 10 mM DTT, and alkylated with 55 mM iodoacetamide. After alkylation, proteins were digested with 6 ng µl^{-1} trypsin (Promega, UK) at 37 °C overnight. The resulting peptides were extracted in 2 % (v/v) formic acid, 2% (v/v) acetonitrile. The digest was analysed by nano-scale capillary LC-MS/MS using an Ultimate U3000 HPLC (ThermoScientific Dionex, San Jose, USA) to deliver a flow of ~300 nl min^{-1}. Peptides were trapped by a C18 Acclaim PepMap100 5 µm, 100 µm x 20 mm nanoViper (ThermoScientific Dionex, San Jose, USA) prior to separation on a C18 Acclaim PepMap100 3 µm, 75 µm x 250 mm nanoViper (ThermoScientific Dionex, San Jose, USA), and eluted with an acetonitrile gradient. The analytical column outlet was directly interfaced via a nano-flow electrospray ionisation source, with a hybrid dual pressure linear ion trap mass spectrometer (Orbitrap Velos, ThermoScientific, San Jose, USA). Data-dependent analysis was carried out, using a resolution of 30,000 for the full MS spectrum, followed by 10 MS/MS spectra in the linear ion trap. MS spectra were collected over a m/z range of 300–2000. MS/MS scans were collected using threshold energy of 35 for collision-induced dissociation. LC-MS/MS data were then searched against a protein database (UniProt KB) with the

Mascot search engine programme (Matrix Science, UK). Database search parameters were set with a precursor tolerance of 5 ppm and a fragment ion mass tolerance of 0.8 Da. Two missed enzyme cleavages were allowed, and variable modifications for oxidized methionine, carbamidomethyl cysteine, pyroglutamic acid, phosphorylated serine, threonine and tyrosine, along with GlyGly and LeuArgGlyGly lysine were included. MS/MS data were validated using the Scaffold programme (Proteome Software Inc., USA). All data were additionally interrogated manually.

SUPPLEMENTARY REFERENCES

1 Liu, Y. T. *et al.* Molecular basis of Wnt activation via the DIX domain protein Ccd1. *J Biol Chem* 286, 8597-8608 (2011).

2 Shatsky, M., Nussinov, R. & Wolfson, H. J. A method for simultaneous alignment of multiple protein structures. *Proteins* 56, 143-156 (2004).

3 Leslie, A. G. The integration of macromolecular diffraction data. *Acta Crystallogr D Biol Crystallogr* 62, 48-57 (2006).

4 Evans, P. Scaling and assessment of data quality. *Acta Crystallogr D Biol Crystallogr* 62, 72-82 (2006).

5 McCoy, A. J., Grosse-Kunstleve, R. W., Storoni, L. C. & Read, R. J. Likelihood-enhanced fast translation functions. *Acta Crystallogr D Biol Crystallogr* 61, 458-464 (2005).

6 Murshudov, G. N., Vagin, A. A. & Dodson, E. J. Refinement of macromolecular structures by the maximum-likelihood method. *Acta Crystallogr D Biol Crystallogr* 53, 240-255 (1997).

7 Emsley, P. & Cowtan, K. Coot: model-building tools for molecular graphics. *Acta Crystallogr D Biol Crystallogr* 60, 2126-2132 (2004).

8 Winn, M. D. *et al.* Overview of the CCP4 suite and current developments. *Acta Crystallogr D Biol Crystallogr* 67, 235-242 (2011).

9 Castaneda, C. A., Kashyap, T. R., Nakasone, M. A., Krueger, S. & Fushman, D. Unique structural, dynamical, and functional properties of k11-linked polyubiquitin chains. *Structure* 21, 1168-1181 (2013).

10 Komander, D. *et al.* Molecular discrimination of structurally equivalent Lys 63-linked and linear polyubiquitin chains. *EMBO Rep* 10, 466-473 (2009).

11 van Dijk, A. D., Fushman, D. & Bonvin, A. M. Various strategies of using residual dipolar couplings in NMR-driven protein docking: application to Lys48-linked di-ubiquitin and validation against 15N-relaxation data. *Proteins* 60, 367-381 (2005).

12 Jung, H. *et al.* Deubiquitination of Dishevelled by Usp14 is required for Wnt signaling. *Oncogenesis* 2, e64 (2013).
